# Lactate and lactylation in cancer

**DOI:** 10.1038/s41392-024-02082-x

**Published:** 2025-02-12

**Authors:** Jie Chen, Ziyue Huang, Ya Chen, Hao Tian, Peiwei Chai, Yongning Shen, Yiran Yao, Shiqiong Xu, Shengfang Ge, Renbing Jia

**Affiliations:** 1 Department of Ophthalmology, Ninth People’s Hospital, Shanghai JiaoTong University School of Medicine, Shanghai, PR China; 2 Shanghai Key Laboratory of Orbital Diseases and Ocular Oncology, Shanghai, PR China; 3 Department of Radiology, Ninth People’s Hospital, Shanghai JiaoTong University School of Medicine, Shanghai, PR China

**Keywords:** Cancer metabolism, Cancer microenvironment

## Abstract

Accumulated evidence has implicated the diverse and substantial influence of lactate on cellular differentiation and fate regulation in physiological and pathological settings, particularly in intricate conditions such as cancer. Specifically, lactate has been demonstrated to be pivotal in molding the tumor microenvironment (TME) through its effects on different cell populations. Within tumor cells, lactate impacts cell signaling pathways, augments the lactate shuttle process, boosts resistance to oxidative stress, and contributes to lactylation. In various cellular populations, the interplay between lactate and immune cells governs processes such as cell differentiation, immune response, immune surveillance, and treatment effectiveness. Furthermore, communication between lactate and stromal/endothelial cells supports basal membrane (BM) remodeling, epithelial-mesenchymal transitions (EMT), metabolic reprogramming, angiogenesis, and drug resistance. Focusing on lactate production and transport, specifically through lactate dehydrogenase (LDH) and monocarboxylate transporters (MCT), has shown promise in the treatment of cancer. Inhibitors targeting LDH and MCT act as both tumor suppressors and enhancers of immunotherapy, leading to a synergistic therapeutic effect when combined with immunotherapy. The review underscores the importance of lactate in tumor progression and provides valuable perspectives on potential therapeutic approaches that target the vulnerability of lactate metabolism, highlighting *the Heel of Achilles* for cancer treatment.

## Introduction

Lactate secretion is widely recognized as a classic metabolic hallmark of cancer, often referred to as the Warburg effect. This phenomenon describes the preference of cancer cells to favor glycolysis for energy production, even in the presence of oxygen, leading to increased lactate production.^1–5^ Recent studies have not only confirmed this understanding but have also delved deeper into the role of lactate in cancer initiation and progression, highlighting its multifaceted contributions beyond merely being a byproduct of cellular metabolism.^6–10^

The traditional view of lactate as a waste product has been challenged by groundbreaking research employing advanced imaging techniques such as ^18^F-fluorodeoxyglucose-positron emission tomography (FDG-PET). A pivotal study by DeBerardinis et al. demonstrated that lactate serves as a vital nutrient for tumor regions, which fundamentally alters the perception of its role in cancer biology.^11,12^ In experiments involving non-small cell lung cancer (NSCLC) xenografts in mice, researchers injected both ^13^C-glucose and ^13^C-lactate. They discovered that metabolites derived from ^13^C-lactate in the tricarboxylic acid (TCA) cycle, such as citrate, glutamate, and malate, were found to be twice as abundant compared to those derived from glucose.^13^ This observation underscores the notion that lactate can serve as a more central and direct substrate in the TCA cycle, a role that has also been corroborated in healthy tissues as well as genetically engineered lung and pancreatic cancer models.^14,15^ Using isotope tracing techniques, scientists have gained insights into the transformation of metabolites within tumors, revealing that lactate is a direct carbon source for the TCA cycle. This innovative approach has allowed researchers to visualize how lactate contributes to the metabolic landscape of tumors, providing a clearer understanding of its role in cancer metabolism.

Abnormal lactate-related metabolisms instigate tumor progression. Lactate metabolism and aberrant glucose and fatty acid metabolism induced by lactate map the metabolic evolution in tumor ecosystem, which coordinates tumor progression.

In the context of tumor cells, the lactate shuttle—facilitating the exchange of lactate between anoxic and aerobic tumor regions—plays a crucial role in tumor surveillance and adaptation to changing metabolic conditions.^16^ Furthermore, lactate impacts both intracellular and extracellular signaling pathways within tumor cells, highlighting its influence on cancer cell behavior and response to therapy.^17^

What’s more, a significant milestone in lactate research is the discovery of lactylation, a post-translational modification that underscores the intersection of metabolism and epigenetics.^18–20^ Lactylation is not limited to histone proteins; it extends to non-histone proteins, thereby influencing a variety of cellular processes. This modification enhances the interaction between metabolic states and epigenetic regulation, accelerating tumor onset, proliferation, metastasis, and the development of drug resistance. Lactate-induced lactylation operates through multiple molecular mechanisms and signaling pathways, often intersecting with other epigenetic modifications to promote a malignant phenotype.^20–22^

**Fig. 1:**
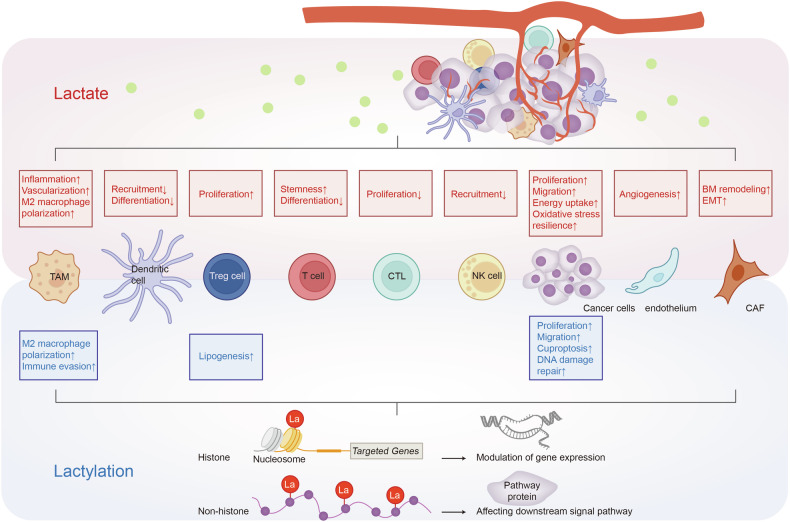
Lactate/Lactylation-targeted therapy stands for the Heel of Achilles for cancer treatment. Lactate/lactylation-targeted therapy mitigates the impact of lactate/lactylation on onco-metabolic reprogramming and tumor microenvironment (TME) remodeling, underscoring the Heel of Achilles for cancer treatment. Generated using Adobe Illustrator (Version 28.2). Abbreviations: BM basal membrane, CAFs cancer-associated fibroblasts; EMT epithelial-mesenchymal transitions; LDHA lactate dehydrogenase A

There is such a fountain of researches related to lactate, which is in vital need of further generalization and summarization.^22–28^ This review straightens out the circuit and physical role of lactate, sorts out the molecular mechanisms of lactate in tumor progression, and describes lactate-targeted strategies of tumor treatment, which strengthens and specifies the potent implementations of lactate in clinical applications and prognosis improvement (Fig. 1).

## Research history of lactate metabolism and lactylation

**Fig. 2:**
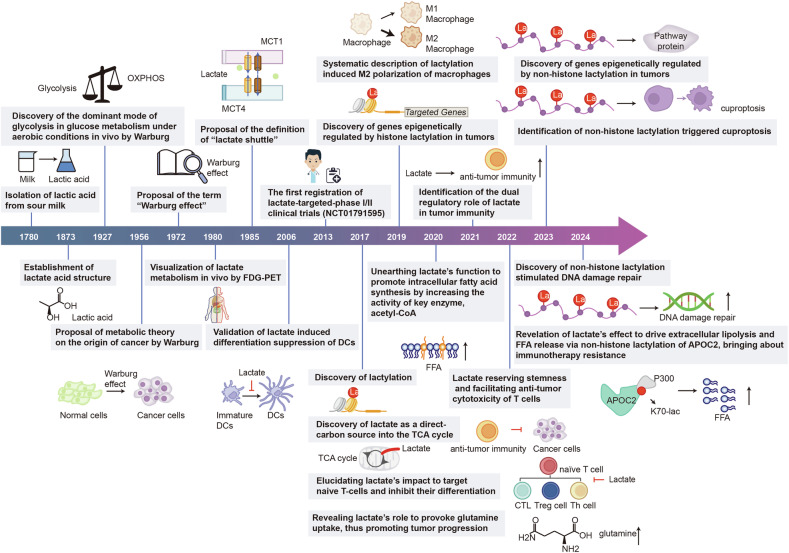
Milestone events of research on lactate metabolism and lactylation. Since lactate was first discovered in 1780, its metabolic role and significance in tumors have gradually been clarified. With advances in isotope-tracing systems, single-cell sequencing, and probe-based metabolic imaging, the biological properties and functions of lactate and lactylation have been extensively explored. Generated using Adobe Illustrator (Version 28.2). Abbreviations: APOC2 apolipoprotein C-II, DCs dendritic cells, FDG-PET ^18^F-fluorodeoxyglucose-positron emission tomography, FFA free fatty acids, K80-lac lysine 80 lactylation, MCT1 monocarboxylate transporter 1, MCT4 monocarboxylate transporter 4

In 1780, the Swedish chemist Carl Wilhelm Scheele was the first to isolate lactic acid from sour milk. Since then, its metabolic role and significance in tumors have gradually been clarified (Fig. 2). Soon, Jöns Jacob Berzelius discovered that lactic acid is also produced by muscles during exercise in 1808. Its structure was established by Johannes Wislicenus in 1873.^29^ Lactic acid is an organic acid with the chemical formula C_3_H_6_O_3_. Lactate is the ionized form of lactic acid. When lactic acid dissolves in water, it can lose a hydrogen ion (proton), resulting in lactate (C_3_H_5_O_3_^−^).^30,31^

Lactate, the end product of glycolysis, was once mischaracterized as a waste since then.^29,32,33^ But recently, there are growing evidence that lactate is a metabolic fuel for skeletal muscle, heart, brain, and malignant cells, that contributes to cell fate decision-making processes.^34–37^ It is also regarded as a metabolic buffer that bridges oxidative phosphorylation (OXPHOS) and glycolysis.^38^

One classic metabolic reprogramming in tumors is the Warburg effect,^36,39–41^ which is associated with lactate production. In 1927, Warburg found that glycolysis remained the dominant mode of glucose metabolism under aerobic conditions in both homozygous mice and human tumor tissues. The Warburg effect was more dominant in malignant tumors compared to benign tumors.^11,32,42–44^ In 1956, He interpreted the mechanism of this phenomenon as a suppressed mitochondrial function and presented the theory that mitochondrial dysfunction underlies tumor aerobic glycolysis as the core of his metabolic theory regarding the origin of cancer.^4,45^ In 1972, researcher Efraim Racker was the first to introduce the term “Warburg effect” to describe the increased glycolytic capacity observed in cancer cells.^46^ However, some studies have challenged the validity of this idea, and the emerging view is that mitochondrial overload leads to an excessive release of lactate.^42,47–50^ In 1980, the adoption of the glucose analogue ^18^F-fluorodeoxyglucose (FDG) in positron emission tomography (PET) could indicate the activity of pyruvate kinase (PK) by tracing glucose metabolism, thus indirectly reflecting the intensity of lactate metabolism through the degree of glycolysis and enabling the quantification of lactate metabolism in vivo.^51–55^ Studies have shown that the conversion of ^13^C carbon from glucose to Krebs cycle intermediates (citrate and succinate) or related metabolites is increased in lung cancer samples compared to non-cancerous paraneoplastic tissues.^56^ Besides, at the single-cell level, it is likely that tumor cells, immune cells, and stromal cells within the TME, which encounter more severe oxygen deprivation, tend to upregulate both glycolysis and mitochondrial OXPHOS.^57,58^ Furthermore, OXPHOS showed a significant correlation with glycolysis in melanoma and head and neck squamous cell carcinoma (HNSCC) as well as with the response to hypoxia.^58,59^ This suggests that the traditional view of tumor cell metabolism under hypoxia, characterized by a switch between glycolysis and mitochondrial respiration, may be inaccurate.^60,61^ Lactate production is not a metabolic driver of cell proliferation and oxidation is the favored metabolic destiny of glucose.

While warburg effect stood for the hallmark of cancer metabolism for about 100 years, recent research as mentioned above have shown that his explanation of the mechanism of aerobic glycolysis is not fully correct.^42,62–65^ Lactate can be produced in significant amounts without impairing aerobic respiration and serves as a direct carbon source into the TCA cycle, which will be further described in chapter 3.2.^13,14^

Additional research has shown that lactate can build a bridge between epigenetics and metabolism through a novel epigenetic modification known as lactylation.^66–70^ Histone lactylation was first documented in 2019 by Zhang et al. the meaning of which was an addition of a lactyl (La) group to the lysine amino acid residues located in the tails of histone proteins.^20^ This study demonstrates the definition of histone lysine lactylation (Kla), a novel form of epigenetic modification, that is observed following the translation of proteins obtained from lactate.

As research on lactate and lactylation in cancer continues to flourish, strategies targeting lactate/lactylation have attracted significant interest as potential anticancer treatments.^71–75^ Consequently, AZD3965, an MCT1 (monocarboxylate transporter 1) inhibitor, has emerged as the first lactate metabolism-targeting drug currently undergoing a phase I/II clinical trial (NCT01791595) for the treatment of advanced solid tumors and non-Hodgkin lymphoma.^76–80^

## Lactate production and transport

When the rate of demand for energy is high, glucose is catabolized and oxidized to pyruvate, which is primarily catalyzed by lactate dehydrogenase (LDH) to produce lactate^81–83^ (Fig. 2). Continuous generation of lactate facilitates the regeneration of NAD+ from NADH.^84,85^ During the reduction of pyruvate to lactate and the oxidation of NADH to NAD+, the NAD+ consumed by the oxidation of glyceraldehyde-3-phosphate in glycolysis is replenished, thus ensuring sustained glycolytic activity and energy production.^86–89^ To prevent lactate accumulation from leading to lactic acidosis, pyruvate dehydrogenase (PDH) catalyzes the production of acetyl coenzyme A from pyruvate, which joins the TCA cycle and irreversibly removes lactate.^84,90,91^ Lactate buildup has the potential to stimulate gluconeogenesis in skeletal muscle and liver cells, converting lactate into glucose and subsequently releasing it into the bloodstream.^92–94^

Lactate is transported in cells via four reversible monocarboxylate transporters (MCT; e.g., MCT1, MCT4).^95–98^ The MCT family achieves lactate exchange across the plasma membrane via H+/lactate cotransport, the direction of which is dependent on the concentration gradient of protons and monocarboxylates.^99–101^ The expulsion of lactate via MCTs removes protons, thereby preserving pH balance within the cytoplasm and inducing acidification in the extracellular environment.^102,103^ Among them, MCT1 is induced by c-Myc to be expressed in all cells and is responsible for the transport of lactate and pyruvate, whereas MCT4 (monocarboxylate transporter 4) is a highly efficient lactate transporter protein induced by hypoxia and is highly expressed in glycolytic tissues (e.g., white muscle fibers) and cancer cells.^26,38,104–106^ Most solid tumors rely on glycolysis for energy production, and upregulation of MCT in cancer contributes to the formulation of an acidic microenvironment, which has a critical impact on cancer cell viability by regulating pH allowing for sustained high rates of glycolysis.^107–109^ Herein, high expression of MCT1/4 in various tumors such as melanoma,^110^ glioblastoma^111^ and NSCLC,^112^ is associated with poor prognosis.

**Fig. 3:**
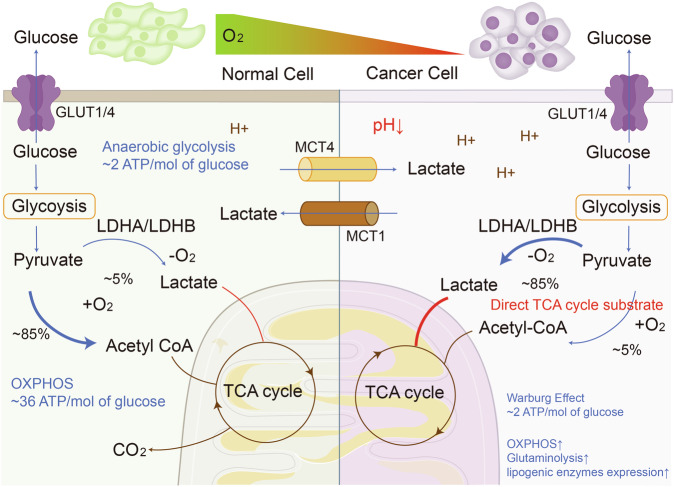
The overview of aberrant tumor lactate-related metabolism compared to physical conditions. In TME, cancer cells exhibit increased tumor glycolysis to meet their high energy demands and metabolic needs. This heightened glycolysis leads to elevated glucose consumption, resulting in excess lactate production and reduced ATP production in the cytoplasm. In normal cells, glycolysis involves ten steps, with the end product pyruvate entering the mitochondria for energy production via the TCA cycle. Besides participating in glucose metabolism, about 10% of the pyruvate is involved in other types of metabolism such as protein metabolism. Failure of pyruvate to enter the TCA cycle leads to decreased energy production compared to glucose molecules through altered glycolysis. Lactate in TME plays a crucial role in regenerating NAD+ molecules and directly join the TCA cycle under hypoxic conditions to sustain glycolysis and ATP production. In addition to glucose metabolism, increased glutaminolysis and lipogenic enzymes expression are also observed in TME. Generated using Adobe Illustrator (Version 28.2). Abbreviations: GLUT1/4 glucose transporter 1/4, TME tumor microenvironment

Lactate shuttle is a concept that was introduced in 1985 and has been continuously developed and refined.^113–116^ The lactate shuttle refers to the transport of lactate between cells, tissues, and organs as a product of glycolysis and a substrate for respiration, which summarizes the process of lactate transmembrane migration and serves as a bridge between anaerobic glycolysis and aerobic respiration.^117,118^ This connection persists under aerobic conditions^29,38,119^ (Fig. 3).

The lactate shuttle performs three physiological functions: 1. lactate is one of the major energy sources. 2. lactate serves as a glucose xenobiotic precursor. 3. lactate is a signaling molecule with autocrine, paracrine, and endocrine-like properties.^29,118,120,121^ The procedure in which muscle-generated lactate is re-transported to the muscle via hepatic glucose isomerization to glucose is known as the Cori cycle.^122–124^ The significance of this cycle is to 1. prevent muscle lactic acidosis under anaerobic conditions, 2. maintain muscle ATP supply, and 3. the Cori cycle stands for a more essential source of substrate for gluconeogenesis than food.^125,126^

Other than intracellular lactate metabolism, lactate may be transported into target cells via nonchannel pathways or MCTs through intercellular shuttling.^127–129^ To date, studies have uncovered that the lactate shuttle is involved in the TME during interactions between various cell populations and this aspect of lactate shuttling is defined as metabolic symbiosis, which is a vital phenomenon of tumor biology.^130,131^

Lactate shuttling between variable cell populations in the TME is a novel finding in oncology, thus revealing the tight association between lactate transport and the progression of tumors.^130,132^ Since lactate serves as a precursor for glycolysis and a substrate for the TCA cycle, the shuttling of this metabolite through TMEs containing both anoxic and aerobic cell populations is of paramount significance. There is an interrelationship and metabolic symbiosis across tumor cells in varied parts of a solid tumor. Because of the rapid growth of tumors, tumor region consist of anoxic and aerobic parts depending on whether they are located in the vicinity of blood vessels. In particular, anoxic tumor cells use lactate dehydrogenase A (LDHA) for lactate anabolism, which enters intercellular matrix through MCT4 and is taken up through MCT1 by aerobic tumor cells. Lactate dehydrogenase B (LDHB) may catalyzes this lactate to pyruvate to generate ATP.^133^ Solid tumor staining is consistent with this perspective. Immunofluorescence staining of pancreatic neuroendocrine tumors showed that MCT4 was predominantly expressed in hypoxic tumor compartments and MCT1 was mostly upregulated in MCT4-negative regions. Metabolic symbiosis invigorates the metabolic potential of tumor tissues for anaerobic environments and facilitates tumor proliferation and metastasis.^130,134,135^ It happens in hypoxic regions of tumor regression and increases invasion and metastasis in mouse models of pancreatic neuroendocrine carcinoma and glioblastoma (GBM).^136^ Studies unveiled that mTOR (mammalian target of rapamycin) mediated lactate shuttle induced by sunitinib/axitinib in PanNET, the inhibition of which significantly reduced tumor burden and viability.^134^

## Multifaceted functions of lactate in cancer cells

**Fig. 4:**
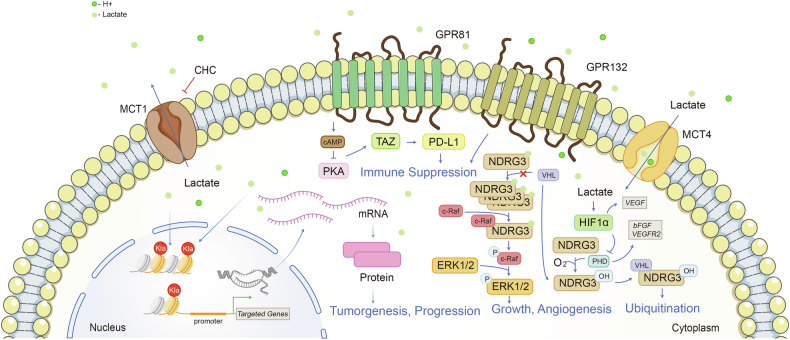
Lactate is an intracellular and extracellular signal transducer of great significance. As it fulfills a role intracellularly, lactate boosts tumor malignancy in hypoxic environments through both HIF-1-dependent and HIF-1-independent pathways. Lactate not only fulfills its function intracellularly, but also serves as an extracellular ligand to GPR81. Extracellularly, GPR81/GPR132 imports lactate and subsequent signaling boosts its utilization, resulting to anti-tumor immunity impairment. Meanwhile, Lactate intensifies the crosstalk between metabolism and epigenetics by editing lactylation modification. Generated using Adobe Illustrator (Version 28.2). Abbreviations: bFGF basic fibroblast growth factor, ERK1/2 extracellular signal-regulated kinase 1/2, GLUT1/4 glucose transporter 1/4, GPR81 G-protein-coupled receptor 81, Kla lysine lactyl; NDRG3, N-Myc downstream-regulated gene family member 3, PHD prolyl hydroxylases, PKA protein kinase A, TAZ transcriptional co-activator with PDZ-binding motif, VHL Von Hippel Lindau tumor suppressor

Lactate has been found to play a crucial role in shaping the TME recently by influencing various cell populations.^137–139^ In tumor cells, lactate amplifies the lactate shuttle, affects cell signaling pathways, enhances oxidative stress resistance, and contributes to lactylation (Figs. 3, 4).

### Circulating carbohydrate fuel of TCA cycle

Organisms rely primarily on OXPHOS and glycolysis to obtain energy from glucose.^140–142^ Traditionally, lactate was considered to be a minor byproduct of glucose utilization in anaerobic environments, but studies now indicate that lactate is an irreplaceable and primary fuel for energy and is crucial for the TCA cycle.^143–146^ In this study, a mouse 3T3-L1 fibroblast based in vitro models indicated that, apart from elevated glucose uptake and lactate release, proliferating fibroblasts showed improvements in mitochondrial respiration as well as coupling efficiency.^147^ Another study showed that pyruvate could not prevent glucose deprivation-induced cell death by facilitating glucose metabolism through gluconeogenesis, suggesting glucose is not one and only substrate for respiration in proliferating cells.^148^ Moreover, lactate is proven to be the main energy donor for the brain, which directly supports energy balance of preopiomelanocortin (POMC) neurons and excitatory neural activity in the brain.^149–151^

Mitochondrial OXPHOS fosters tumorigenesis, development, metastasis, and drug resistance.^152–156^ Pyruvate migrates into mitochondria during OXPHOS to engage in the TCA cycle.^157,158^ While research on the Warburg effect laid the foundation for the role of lactate in the TME through the glycolytic pathway, recent studies have revealed that lactate could be utilized as a carbon origin directly through the TCA cycle in tumor cells (Fig. 3). Isotope tracing showed that TCA metabolites labeled by ^13^C-lactate continued to outcompete that with ^13^C-glucose label in a mouse tumor model even in the presence of glucose.^14^ This reverses previous perceptions and establishes that lactate is a proximate fuel for the TCA cycle. Utilization of lactate by TCA cycle even correlates with the metastatic capacity of the tumor. Isotopic tracing of xenografts in mice showed that MCT1-mediated lactate utilization, reflected by TCA metabolites, was elevated in tumors with high metastatic potential compared with tumors with low metastatic potential.^16^ Other articles have also emphasized this emerging perspective that glucose serves as a specific fuel while lactate as a universal fuel.^159,160^ In a word, these studies illustrate that lactate is by no means just a useless byproduct of rapid tumor energy consumption in anaerobic stress. It is a direct and vital participant in the TCA cycle and OXPHOS.

### Buffer of redox homeostasis

Lactate serves as a vital metabolic substrate and functions as an intercellular and intertissue redox signaling molecule.^161,162^ First, lactate production and removal uphold electron flux through a specific pathway, involving the conversion of NADH to NAD+ and H+ alongside LDH-mediated conversion of lactate to pyruvate.^163^ These reduced coenzymes (nicotinamide adenine dinucleotides (NAD+ or NADP+)) produce electrons as they undergo oxidation via either mitochondrial respiration or lactate fermentation, thereby sustaining redox balance.^164^ Besides, as a modulator of OXPHOS, lactate affects intracellular redox homeostasis.^165^ Last but not least, lactate benefits redox condition maintenance. Downregulation of MCT expression leads to loss-function of LDH, intracellular accumulation of lactate, cytoplasmic acidification, and cell death in cancer cells.^166^

### Regulator of amino acid and fatty acid metabolism

As a specific feature of the process of lactate metabolism in cancer cells, glutamine provides a carbon source and promotes the utilization of lactate and TCA cycle intermediates. Regulated by cellular myelocytomatosis (c-Myc), glutamine is transported across cell membrane by amino acid transporter type 2 (ASCT2) and sodium-coupled neutral amino acid transporter 5 (SN2). Glutaminase (GLS/GLS2) catalyzes it and transforms it to glutamate. Next, glutamine enters the TCA cycle as α-ketoglutarate (α-KG). Via this above-mentioned pathway, glutamine becomes the second-largest carbon source of lactate in cancer cells.^167^ Additionally, lactate induces the expression of the proto-oncogene c-Myc. c-Myc transcriptionally binds to the promoter region of glutamine importers, ASCT2 and SN2, leading to increased glutamine uptake and tumor progression.^168–170^

Fatty acid metabolism fosters tumorigenesis, progression, and treatment resistance through enhanced lipid synthesis, storage, and catabolism.^171,172^ It is well known that lactate accumulation can promote intracellular fatty acid synthesis by promoting the activity of acetyl coenzyme A carboxylase (ACC), a key enzyme in fatty acid synthesis, and by supplementing the raw material for fatty acid synthesis, acetyl coenzyme A (acetyl-CoA).^173,174^ Accumulation of lipid droplets in the cytoplasm of cancer cells is correlated with cancer invasiveness and chemotherapy resistance.^175,176^ The expression of lipogenic enzymes is upregulated and their activity is on the rise in most tumors. For instance, citrate lyase is an indispensable modulator of histone acetylation in cancer cells.^177^ Lactate promotes intracellular fatty acid synthesis by supplementing acetyl-CoA, a raw material for fatty acid synthesis, and by increasing the activity of acetyl-CoA carboxylase, a key enzyme in fatty acid synthesis.^178^ Besides, as lactate acts as a favored energy source for muscle and heart cells through OXPHOS, it simultaneously inhibits lipolysis and blocks the import of free fatty acids (FFA) into mitochondria via carnitine palmitoyltransferase 1 (CPT1).^179,180^ Nonetheless, through the integration of multi-omics analysis and validation both in vitro and in vivo in NSCLC, a recent study disclosed that intracellular lactate drives extracellular lipolysis and FFA release via non-histone lactylation of apolipoprotein C-II (APOC2), bringing about immunotherapy resistance.^181^ These studies show that role of lactate in fatty acid metabolism differs between the TME and normal tissues, suggesting a complexity in its function. The particular signaling pathways by which lactate impacts fatty acid metabolism and their significance in tumor progression remain to be further explored.

### Intracellular and extracellular signaling of tumor cells

Lactate is a predominant signal transducer of tumor cells (Fig. 4). Lactate inhibits 2-oxoglutarate-dependent prolyl hydroxylases (PHDs) (mainly PHD2), which in turn prevents Von Hippel Lindau tumor suppressor (VHL)-mediated ubiquitination of hypoxia-inducible factor 1 (HIF-1) and its proteasomal degradation, thus stabilizing HIF-1. Lactate-mediated PHD2 damage relies on the oxidation of lactate to pyruvate, which elicits suppression of PHD2 through pyruvate binding, alongside HIF-1-mediated elevation of *VEGF*.^182^

In addition, lactate induces angiogenesis and maintains tumor metabolism in hypoxic environments by inhibiting the PHD2/VHL system through an HIF-1-independent pathway that cooperates with the HIF-1 pathway. Lactate directly binds to N-Myc downstream-regulated gene family member 3 (NDRG3; NM_032013) protein and inhibits its binding to PHD2 and its deterioration, securing prolonged protein stability. Accumulation of NDRG3 leads to activation of Raf/ERK-mediated angiogenesis and proliferation, bringing about cellular adaptation to long-term hypoxia. Animal experiments showed that knockdown of NDRG3 inhibited the proliferation of hepatocellular carcinoma (HCC) subcutaneous tumors and angiogenesis of subcutaneous stromal plugs. Downregulation of lactate metabolism inhibited the proliferation of lymphoma cells at the cellular level and in subcutaneous tumors, and overexpression of NDRG3 reversed the growth inhibition caused by downregulation of lactate metabolism.^17^

Nevertheless, lactate, as a redox homeostasis regulator, can act as an antioxidant to resist excessive oxidative stress in tumor cells and reverse cellular DNA/RNA damage caused by massive reactive oxygen species (ROS) production, thus mediating treatment resistance and metastasis.^162,183^ Dou et al. unearthed that lactate enhanced the production of ROS through nicotinamide adenine dinucleotide phosphate oxidase 1 (NOX1), which induced the senescence-associated secretory phenotype (SASP). In contrast, inhibiting pyruvate dehydrogenase kinase 4 (PDK4) mitigates lactate-induced DNA damage and curbs the SASP.^184–187^ In addition, Hu et al. confirmed that in 4T1 and HeLa cells, LDHA mediated hydrogen peroxide production under oxidative stimuli in vivo and in vitro.^188^ Concerning cervical tumor, nuclear LDHA acquired a non-canonical enzymatic activity to produce α-hydroxybutyrate (α-HB), which facilitated interaction between disruptor of telomeric silencing 1-like (DOT1L) and LDHA, which mediated hypermethylation of histone H3K79. This process led to the activation of antioxidant genes and enhanced Wnt signaling pathway, thus promoting tumor growth.^189^ In patients with NSCLC, elevated LDHA expression is a negative prognostic factor linked to radiation resistance. Inhibition of LDHA by oxamate significantly boosted radiosensitivity and enhanced apoptosis, autophagy and cell cycle turbulence triggered by ionizing radiation (IR) in A549 and H1975 cancer cells.^190^ Response to fractionated irradiation correlates with lactate concentration of tumor regions in 10 xenografted human HNSCC tumor lines.^191^ Besides, disturbances in oxidative homeostasis due to lactate are also associated with metastasis. Studies elucidated that the heterogeneity of the metastatic process in melanoma depends on the differences in the expression levels of MCT1, and revealed that the molecular mechanism involved lies in the fact that melanomas with high expression of MCT1 can utilize lactate to resist oxidative stress, and thus obtain a stronger metastatic capability.^13,16^

Apart from its character as a signaling mediator intracellularly, meanwhile, lactate functions as an extracellular ligand.^192–196^ G-protein-coupled receptor 81 (GPR81), a G protein-coupled receptor for lactate, exists in colon, breast, lung, hepatocellular, salivary gland, cervical, and pancreatic cancer cell lines.^197–200^ Lactate supports energy metabolism in tumor cells through binding to GPR81. In pancreatic cancer samples, 94% (148/158) of patients expressed high levels of GPR81. Functionally, knockdown of GPR81 in lactate-containing low-glucose culture conditions resulted in decreased mitochondrial activity and massive death of pancreatic cancer cells. The addition of lactate to the culture medium induced the expression of genes involved in lactate uptake and metabolism, but not in GPR81-silenced cells. Under conditions that mimicked the TME (low glucose, glutamine, and pyruvate), the levels of MCT1, MCT4, cluster of differentiation 147 (CD147), peroxisome proliferator-activated receptor γ coactivator 1 α (PGC-1α) and other mRNAs were increased after 6 h of lactic acid treatment in parental pancreatic cancer cells expressing GPR81. In contrast, lactate treatment had no effect on the mRNA levels of these molecules mentioned above after GPR81 silencing. In addition to altering mitochondrial activity, mouse in situ pancreatic tumor models constructed by shGPR81 cell line had slower tumor growth, longer overall survival, and slower lung metastasis. In conclusion, GPR81-lactate transport is an important cancer cell transporter mechanism, which promotes energy consumption, proliferation and metastasis of pancreatic cancer.^201^ In TME, this pathway induces immunosuppression. In lung cancer cells, activation of GPR81 decreases intracellular cyclic adenosine monophosphate (cAMP) levels and inhibits protein kinase A (PKA) activity, leading to activation of Transcriptional co-activator with PDZ-binding motif (TAZ), which further activates the programmed cell death protein 1/programmed death-ligand 1 (PD-L1/PD-1) immune checkpoint pathway and impairs T-cell function.^202,203^ In addition to GPR81, G-protein coupled receptor G2A (GPR132) is an essential transmembrane lactate receptor which leads to immune suppression and metastasis as well.^204,205^ Lactate activated Gpr132 on macrophage, which facilitates M2 polarization and promoted adhesion, migration, and invasion of breast cancer and lung cancer.^206,207^ In vivo and in vitro experiment of colorectal cancer (CRC) clarified platelet reactive protein 2 (THBS2) induced HIF-1α/lactate/GPR132 pathway promoted M2 polarization of macrophages, resulting in inhibition of T-cell proliferation and cytotoxicity.^208^ In conclusion, lactate acts as an extracellular ligand and an intracellular signal transduction factor to facilitate the energy uptake, proliferation, migration, and immune escape processes of tumor cells.

## Lactylation serves as the bridge between metabolism and epigenetics

**Fig. 5:**
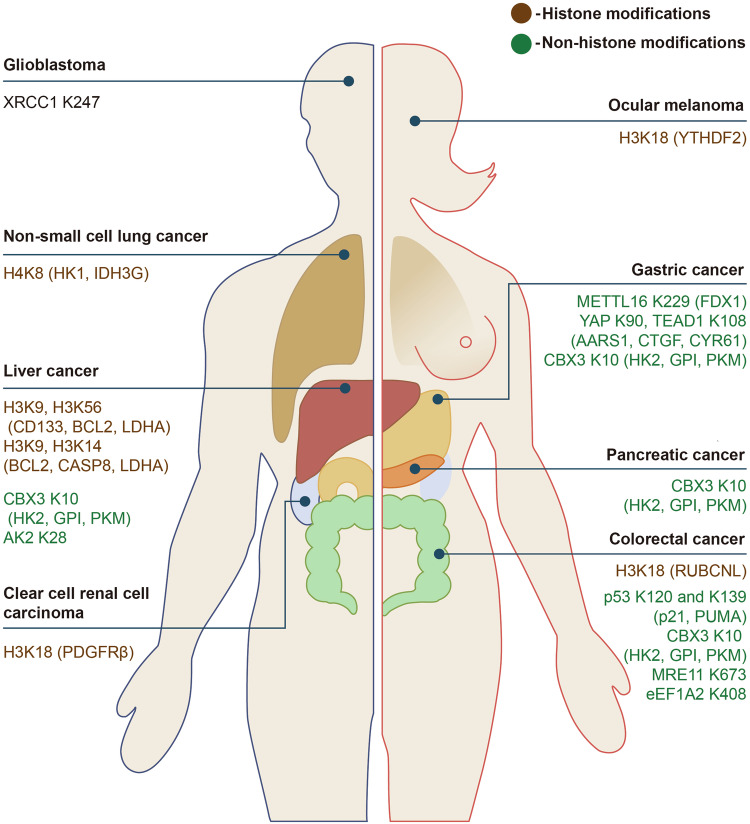
Histone/non-histone lactylation sites and their downstream genes following modification. Histone and non-histone lactylation sites and their downstream genes after modification are presented in the form of lactylation sites (downstream genes), with histone lactylation shown in brown and non-histone lactylation shown in green. Generated using Adobe Illustrator (Version 28.2). Abbreviations: AARS1, alanyl-tRNA synthetase 1; AK2 adenylate kinase 2, BCL2 B-cell lymphoma 2, CASP8 caspase 8, CBX3 chromobox 3, CD133 cluster of differentiation 133, CTGF connective tissue growth factor, CYR61 cysteine-rich protein 61, eEF1A2 elongation factor 1 alpha 2, FDX1 ferredoxin 1, GPI glucose-6-phosphate isomerase, HK1 hexokinase 1, HK2 hexokinase 2, IDH3G isocitrate dehydrogenase (NAD+) 3 gamma, LDHA lactate dehydrogenase A, METTL16 methyltransferase Like 16, MRE11 meiotic recombination 11, p21 p21^CIP1/WAF1, PDGFRβ platelet-derived growth factor receptor β, PKM pyruvate kinase M, PUMA p53 upregulated modulator of apoptosis, RUBCNL rubicon like autophagy enhancer, TEAD TEA domain transcription factor, XRCC1 X-ray repair cross-complementing 1, YTHDF2 YTH N (6)-methyladenosine RNA binding protein 2

Lactate intensifies the crosstalk between metabolism and epigenetics^18–20,209,210^ (Fig. 5). Recently, there have been histone and nonhistone aspects of Kla.^157,211–214^ Interestingly, numerous research have demonstrated the buildup of histone Kla on the genome in cytoplasm triggered by hypoxia, interferon-γ (IFNγ), lipopolysaccharide (LPS), or bacterial infection, bringing about lactate production.^20,215^

Besides, Kla epigenetically regulates gene expression.^216–219^ Supplementation of exogenous sodium lactate (NaLa) to B-cell adapter for PI3K (BCAP)-deficient bone marrow-derived macrophages (BMDM) reverses the downregulated Arginase-1 (ARG1) and Krüppel-like factor 4 (KLF4) expression caused by BCAP deficiency.^215^ ChIP assay of lactate-treated BMDM reveals significant up-regulation of histone Kla in the promoter region of the *ARG1, platelet-derived growth factor A (PDGFA)*, *thrombospondin-1 (THBS1)*, and *vascular endothelial growth factor A (VEGFA)*.^220^ It follows that Kla regulates gene expression of immune cells and plays an essential physiological role.

Histone Kla has been sequentially reported in a variety of cancers,^221–224^ especially histone H3 lysine 18 lactylation (H3K18la). H3K18la has been reported to play a part in multitudinous biological processes such as oncogenesis,^221,225–227^ progression,^228,229^ tumor immune escape^28^ and cancer cellular metabolism reprogramming^21^ (Figs. 4, 5). Yang et al. revealed that inactivated VHL upregulated H3K18la, which promoted the expression of platelet-derived growth factor receptor β (PDGFRβ) and formed a positive feedback loop, thereby promoting the proliferation and metastasis of clear cell renal cell carcinoma (ccRCC). Li et al. lately revealed that in CRC cells, high H3K18la level promoted the transcription of Rubicon-like autophagy enhancer (RUBCNL/Pacer), which enhanced autophagy through promoting autophagosome maturation, and contributed to CRC tumorigenesis and progression.^230^ Yu et al. elucidated histone Kla levels were greater in ocular melanoma than in normal tissue and were positively correlated with poor prognosis in patients. Additional exploration of potential mechanisms indicates a facilitated expression of YTH N (6)-methyladenosine RNA binding protein 2 (YTHDF2) in ocular melanoma cells, resulting from elevated Kla of its promoter. In addition, YTHDF2, as an N6-m6A reader, recognizes m6A-modified period circadian regulator 1 (PER1) and tumor protein p53 (TP53) mRNAs and promotes their degradation, thereby accelerating ocular melanoma tumorigenesis.^221^

In addition to promoting tumor proliferation, metastasis, and invasion by modulating the expression of epigenetically related genes in tumor cells, Kla is also capable of inducing the expression of TCA cycle-related enzymes.^231,232^ A global lactylome profiling of cancer and paracarcinoma tissues from patients with hepatitis B virus-related HCC (HCC) successfully identified lactylation modification sites located on both non-histone and histone proteins. It suggests that lactylation modification may be involved in a broader biological function besides transcriptional regulation. More importantly, Kla preferably affects enzymes involved in metabolic pathways, including glucose metabolism, TCA cycle, amino acid metabolism, fatty acid metabolism, and nucleotide metabolism, and that elevated Kla levels on metastasis-related substrate are strongly correlated with aggressive clinical features and driver mutations of HCC.^21^ Studies of NSCLC have shown that histone Kla leads to downregulated level of the glycolysis-related enzymes and concurrently elevated that of the TCA cycle-related enzymes.^222^ In summary, Kla improves glucose uptake by tumor cells by modifying the expression of metabolism-related genes and adds up to metabolic disorders of NSCLC.

Besides, Kla incorporates target therapy resistance.^233,234^ Scientists demonstrated downregulation of histone Kla enhanced the sensitivity of CRC cells to bevacizumab treatment in cell-based xenografts, patient-derived xenografts and patient-derived organoids models, which further broadens the role of Kla in antiangiogenic therapy.^230^

Regarding non-histone lactylation, other research also validated its effect on prompting tumor progression.^235–238^ Zong et al. uncovered that alanyl-tRNA synthetase 1 (AARS1) detected lactate and facilitated the site-specific lactylation of p53, which weakened its ability to bind DNA and underwent liquid-liquid phase separation (LLPS). As a result, tumor-suppressing functions of p53 were diminished in a CRC mouse model.^239^ Lysine acetyltransferase 8 (KAT8), a lysine acetyltransferase known for its pan-Kla writing capabilities, catalyzes the lactylation of elongation factor 1 alpha 2 (eEF1A2) at lysine (Κ)-408 in CRC, which promoted tumorigenesis.^240^ Besides, lactate leads to the Kla of methyltransferase-like 16 (METTL16)-K229, which further induces m6A modification of ferredoxin 1 (FDX1) mRNA, increases FDX1 mRNA expression, and ultimately leads to the death of gastric cancer cells via cuproptosis.^241^ Yang et al. discovered adenylate kinase 2 (AK2) K28 lactylation enhanced proliferation and metastasis of HCC cells.^21^ Furthermore, global lactylome profiling of gastrointestinal (GI) cancers, including liver, pancreatic, colorectal, and gastric cancers, further demonstrated that non-histone lactylation exhibits cross-talk with various forms of epigenetic regulation.^21^ Lactylation of chromobox 3 (CBX3) at K10 facilitates its binding to H3K9me3, which in turn drives the invasiveness of GI cancers.^242^

Moreover, similar to histone lactylation, non-histone lactylation modifications play a role in tumor treatment resistance. Chen et al. found that non-histone lactylation of meiotic recombination 11 (MRE11) boosted homologous recombination (HR) and chemoresistance in CRC.^243^ Li demonstrated that the interaction between aldehyde dehydrogenase (ALDH) 1 family member A3 (ALDHA3) and pyruvate kinase M2 (PKM2) increased lactate production, which in turn induced lactylation of XRCC1 at K247. This enhanced DNA damage repair and resulted in resistance to both radiotherapy and temozolomide (TMZ)-based chemotherapy.^244^ Nevertheless, recent reports have revealed the potential therapeutic effect of histone/non-histone lactylation in tumors, which will be further depicted in “Other lactate-targeted strategies”.^245^ In addition to lactylation, lactate is involved in regulating other epigenetic modifications that foster tumor progression. The Warburg effect results in a dramatic increase in intracellular levels of acetyl coenzyme A (Ac-CoA), which induces general control non-derepressible 5 protein/Spt-Ada Gcn5-acetyltransferase (Gcn5p/SAGA)-catalyzed acetylation of histone proteins, which induces downstream gene transcription and promotes cell growth.^246,247^ Apart from acetylation, lactate alters the anaphase-promoting complex (APC/C) by directly blocking the Small Ubiquitin-like Modifier protease (SUMO protease) sentrin/SUMO-specific protease 1 (SENP1) and stabilizing SUMOylation at two specific residues on APC4. The stabilization of SUMOylation induced by lactate accelerates the degradation of cell cycle proteins and ensures effective mitotic exit in actively dividing human cells.^248^

Previous studies have explored whether lactylation and acetylation have functional overlap.^249^ Their similarity lied in the fact that lactate and acetyl-CoA both were primarily derived from the glycolytic end product, pyruvate and had similar molecular structures.^231,250^ Besides, from the perspective of modification mechanisms, both lactylation and acetylation preferred targeting lysine (Lys) residues for epigenetic modulation, which utilized p300 as the “writer” for Lys catalysis and class I-III histone deacetylases (HDAC1-3) as “eraser”.^251–255^ However, recent studies have firmly established that lactylation functioned differently from acetylation. In 2019, Utilizing M1 macrophages exposed to bacteria as a model system, Zhang et al. reveal that histone lactylation follows a different temporal pattern compared to acetylation.^20^ Then, it was elucidated that lactylation exhibited slower kinetics at lysine compared to acetylation, and intracellular concentrations of lactyl-CoA were lower than those of acetyl-CoA.^105^ Recently, Zong et al. utilized a clustered regularly interspaced short palindromic repeat (CRISPR) screen, which identified AARS1 as an intracellular sensor for lactate and a transferase of lactyl to lysine residues. AARS1 binds directly to lactate, catalyzes the adenosine triphosphate (ATP)-dependent synthesis of lactate-AMP and mediates widespread lysine lactylation, including that of p53.^239^ AARS1 was also uncovered to lactylate and activate the Yes-associated protein-TEA domain transcription factor (YAP-TEAD) complex in gastric cancer. As a Hippo target gene that creates a positive-feedback loop with YAP-TEAD complex, AARS1 promotes proliferation of gastric cancer.^256,257^ Additionally, alanyl-tRNA synthetase 2 (AARS2) has been shown to act as a mitochondrial lactyltransferase as well, catalyzing the lactylation of K336 in pyruvate dehydrogenase complex 1 (PDHA1) and K457/8 in carnitine palmitoyltransferase 2 (CPT2) under hypoxic conditions. This lactylation process mediates mitochondrial proteins to regulate OXPHOS in muscle cells.^258^ Moreover, recent studies have provided deeper insights into the role of AARS1/2 in the transferase-catalyzed lactylation process, revealing that lactate is directly modified onto proteins via catalysis, eliminating the need for lactoyl-CoA formation.^259^ As AARS1/2 is not a pan-Kla writer, to summarize, these results unearth that lactylation and acetylation involve different enzymatic toolkits.

## Lactate and lactylation in diverse cell populations

Deep-in-depth investigation of the TME complexity and collaborations between the abundant cell types within this niche stands for a stepping stone to precision cancer therapy.^260–262^ According to the fascinating and challenging features of TME, it consists of multiple populations of fibroblasts, an underdeveloped vascular system, and a varied and predominantly suppressive array of immune cells.^263–265^ The invasiveness, resistance to therapy, and heterogeneity of the tumors are therefore influenced to a significant extent by the non-malignant parts of the tumor, in addition to tumor cells themselves.^266–268^

**Fig. 6:**
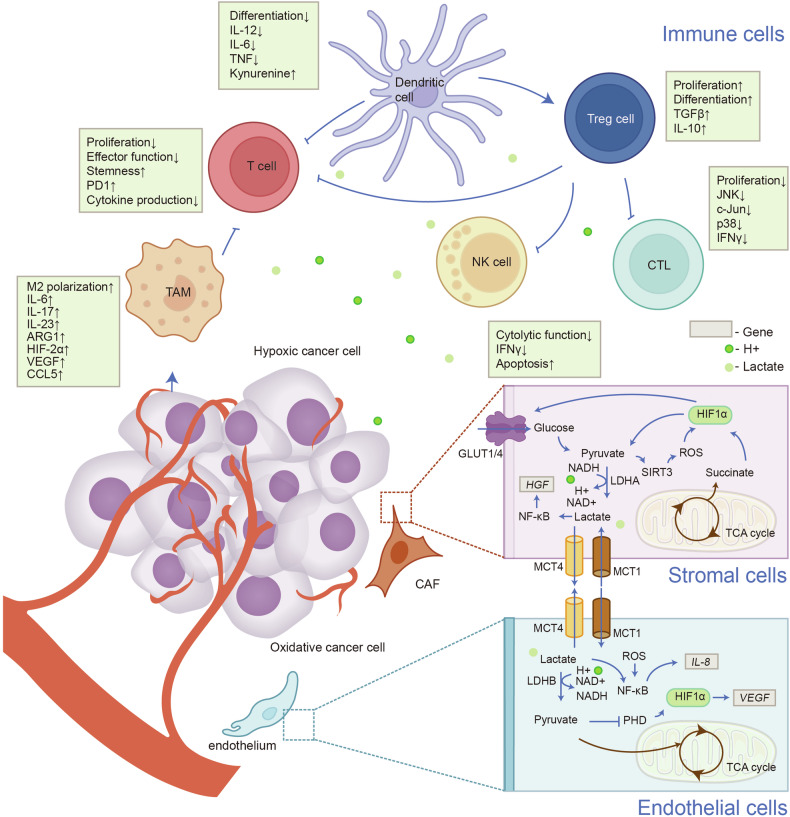
Lactic acid remodels variant cell populations in the TME. The TME consists of various cell types, including tumor, stromal, endothelial, and immune cells. Lactate impacts infiltrating immune cells by regulating their metabolism due to the Warburg effect, inhibiting the activation and proliferation of CD8 + T cells, natural killer (NK) cells, and dendritic cells, while promoting the immunosuppressive function of CD4 + CD25+ regulatory T (Treg) cells. Lactate also aids the polarization of macrophages towards an anti-inflammatory (M2-like) phenotype, supporting angiogenesis, tissue remodeling, and tumor progression. In cancer associated fibroblasts (CAFs), lactate production, driven by SIRT3/succinate-dependent HIF-1α activation, enhances BM remodeling, EMT, metastatic reprogramming, and treatment resistance. In endothelial cells, LDHB converts lactate to pyruvate, which enters the TCA cycle, influencing redox status, inducing reactive oxygen species (ROS), stabilizing HIF-1, and activating NF-κB signaling, which increases *IL-8* and *VEGF* transcription. Thus, lactate significantly favors tumor progression, though detailed mechanisms remain unclear. Generated using Adobe Illustrator (Version 28.2). Abbreviations: bFGF, basic fibroblast growth factor; ERK1/2, extracellular signal eegulated kinase 1/2; GPR81, G-protein-coupled receptor 81; PHD, prolyl hydroxylases; PKA, protein kinase A; RUBCNL, Rubicon like autophagy enhancer; TAZ, transcriptional co-activator with PDZ-binding motif; VHL, Von Hippel Lindau tumor suppressor

In other cell populations, on the one hand, interaction between lactate and immune cells exerts an effect on impaired cell differentiation, reduced immune response, evasion of immune monitoring, and defective sensitivity upon treatment.^269,270^ On the other hand, lactate/lactylation crosstalk with stromal/endothelial cells reinforces basal membrane (BM) remodeling, epithelial-mesenchymal transitions (EMT), metabolism reprogramming, angiogenesis, and drug resistance (Fig. 6).

### Immune cells

Lactate/lactylation induces the generation of immunosuppressive TME in diverse types of tumors^271–275^ (Table 1). Xiong et al. elucidated that lactylation of methyltransferase like 3 (METTL3) was upregulated in tumor-infiltrating myeloid cells (TIMs), thus inducing immunosuppression CRC.^28,276^

**Table 1:** Functions of lactate and lactylation in tumor immunity

Immune cell type	Mechanism	Regulated gene	Cancer type	Function	Ref
TIMs	non-histone modifications (METTL3 K281, K345)	JAK1	CRC	induce immunosuppression and tumor immune evasion	^28^
macrophages	histone modifications (H3K18)	Arg1, Vegfa	Breast cancer	drive M2 macrophage polarization	^20^
macrophages	lactate metabolism	Arg1	LLC	induce ACLY-dependent H3K9ac, thereby upregulating the expression of M2-like genes.	^287^
macrophages	lactate metabolism	Arg1, Vegfa	Melanoma, LLC	drive M2 macrophage polarization and promote tumor growth	^283^
macrophages	lactate metabolism	Gpr132, CD206	Breast cancer	drive M2 macrophage polarization through Gpr132, thereby enhancing lung metastasis and invasiveness	^206^
macrophages	lactate metabolism	ATP6V0d2, HIF-2α	LLC	promote HIF-2α induced tumor vascularization and growth	^284^
macrophages	lactate metabolism	ERK1/2, STAT3	Breast cancer	drive M2 macrophage polarization	^286^
macrophages	lactate metabolism	SREBP2	Breast cancer	Downregulation of LDHB skews TAMs to function as a lactate and sterol/oxysterol source for the proliferation	^289^
macrophages	lactate metabolism	IL-23	LUAD	promote inflammation and tumor development.	^288^
microglia	lactate metabolism	IGFBP6, ARG1, CD206, CD163	GBM	drive M2 microglia polarization	^290^
macrophages	histone modifications (H3K18)	RARγ	CRC	enhance tumorigenesis through prohibiting RAΡγ expression and inducing IL-6 production, which activated STAT3 in tumor cells	^294^
DCs	lactate metabolism	IFN-α, IFN-γ	LLC	Inhibit DCs from presenting antigens and activating CD8 + T cells, preventing them from initiating an immune response within tumors in vivo	^281^
DCs	lactate metabolism	IFN-α	Breast cancer	weaken IFNα induction and reduce the recruitment Treg cells	^297^
DCs	lactate metabolism	CD1a, CD80, CD86, CD16	Urothelial carcinoma, melanoma, prostate carcinoma	block the differentiation of TADC and reduce their IL-12 secretion	^278^
DCs	lactate metabolism	SREBP2	Melanoma	transform conventional DCs into CD63+ mregDCs through homeostatic or tolerogenic maturation	^280^
Treg cells	lactate metabolism	PD-1	CRC, melamoma, NSCLC, gastric cancar, AML	promote PD-1 expression in Treg cells, whereas dampening PD-1 expression by effector T cells	^298,299^
Treg cells	lactate metabolism	LDHA, SLC16A1	Melanoma, HNSCC	counteract the destabilizing impact of high-glucose conditions towards Treg cells, thus promoting their proliferation and immunosuppressive function	^300^
Treg cells	non-histone modifications (APOC2 K70)	APOC2	NSCLC	produce FFA, recruit Treg cells and elicit immunotherapy resistance	^300^
NK cells, CD8^+^ T cells	lactate metabolism	IFN-γ, granzyme B	Melanoma	down-regulated LDHA promotes inflitration and enhances the therapeutic effect of anti-PD-1 therapy	^302^
CTL	lactate metabolism	JNK, c-Jun, p38	RCC	suppress CTL n a manner of immediate onset and reversion.	^306^
MDSCs	lactate metabolism	PKM2, LDHA	Pancreatic cancer	bolster MDSCs and promote a suppressive immune microenvironment	^314^
T cells	lactate metabolism	FIP200	Ovarian cancer	targeting naive T-cells and inhibit FIP200 expression to evade the immune response	^307^
T cells	lactate metabolism	TCF1	Melanoma, CML	block one-carbon metabolism, decrease H3K27me3 deposition of memory-related genes, thereby affecting differentiation, reserving sternness and facilitating anti-tumor cytotoxicity of T cells	^318^
T cells	lactate metabolism	TCF7, TCF1	CRC	enhance H3K27ac at the TCF7 super-enhancer locus and the stemness of CD8 + T cells	^316^
CD8 + T cells	lactate metabolism	IFNγ, IL-2, TNFα	Breast cancer, CRC	reduce tumor growth, with the effect being dependent on T cells	
CD4+/8 + T cells	lactate metabolism	IFN-γ, granzyme B	Melanoma, LLC, colon adenocarcinoma	stimulate the production of antitumor cytokines and reduce tumor growth, with the effect being dependent on T cells	^315^

Abbreviations: *ACLY* adenosine triphosphate–citrate lyase, *AML* acute myeloid leukemia, *APOC2* apolipoprotein C-II, *Arg1* arginase-1, *ATP6V0d2* ATPase H+ transporting V0 subunit d2, *CD16* cluster of differentiation 16, *CD163* cluster of differentiation 163, *CD1a* cluster of differentiation 1a, *CD206* c-type lectin domain family 10 member A, *CD63* cluster of differentiation 63, *CD80* cluster of differentiation 80, *CD86* cluster of differentiation 86, *c-JUN* Jun proto-oncogene, *CML* chronic myeloid leukemia, *CRC* colorectal cancer, *DCs* dendritic cells, *ERK1/2* extracellular signal regulated kinase 1/2, *FFA* free fatty acids, *FIP200* FAK family kinase-interacting protein of 200 kDa, *Gpr132* G-protein coupled receptor G2A, *H3K18* histone H3 lysine 18, *HNSCC* head and neck squamous cell carcinoma, *IGFBP6* insulin-like growth factor-binding protein 6, *LDHA* lactate dehydrogenase A, *LDHB* lactate dehydrogenase B, *LLC* Lewis lung carcinoma, *LUAD* lung adenocarcinoma, *MDSC* myeloid-derived suppressor immune cells, *METTL3* methyltransferase like 3, *mregDCs* mature regulatory DCs, *NSCLC* non-small cell lung cancer, *PKM2* pyruvate kinase M2, *RARγ* retinoic acid receptor γ, *RCC* renal cell carcinoma, *SLC16A1* solute carrier family 16 member 1, *SREBP2* sterol regulatory element-binding protein 2, *STAT3* signal transducer and activator of transcription 3, *TADC* tumor-associated dendritic cells, *TCF1* T cell factor 1, *TCF7* T cell factor 7, *TIMs* tumor-infiltrating myeloid cells

Lactate represses differentiation and antigen presentation of dendritic cells (DCs).^117,277^ Previous study discovered that cocultures of melanoma and prostate carcinoma multicellular tumor spheroids (MCTSs) produced low levels of macrophage colony-stimulating factor (MF-CSF) and interleukin-6 (IL-6), while generating significant amounts of lactic acid. Furthermore, introducing lactic acid in the process of DCs differentiation in vitro led to a phenotype similar to that of tumor-associated dendritic cells (TADCs) formed within melanoma and prostate carcinoma MCTSs, marked by inhibited differentiation and reduced IL-12 secretion.^278,279^ Plebanek et al. discovered that lactate from melanoma stimulates sterol regulatory element-binding protein 2 (SREBP2) in tumor DCs, leading to the transformation of conventional DCs into cluster of differentiation 63 (CD63)+ mature regulatory DCs (mregDCs) through homeostatic or tolerogenic maturation. Targeted genetic silencing of SREBP2 in DCs, as well as its pharmacologic inhibition, enhanced antitumor CD8 + T cell activation and inhibited melanoma progression.^280^ Moreover, Caronni revealed that lactate inhibited DCs from presenting antigens and activating CD8 + T cells when co-cultured with Lewis lung carcinoma (LLC) cells, thus preventing them from initiating an immune response within LLC models in vivo.^281^ Furthermore, Nasi et al. uncovered that the lactate-induced changes in DCs might be density-dependent. In dense cultures, disrupting lactate production revealed its key role in reshaping DC functions, leading to increased production of interleukin-12 (IL-12) and decreased interleukin-10 (IL-10). However, in sparse cultures, the effects were reversed.^282^

Pertaining to microglia/macrophages, lactate and histone lactylation promote the conversion of macrophages to M2-type tumor-associated macrophages (TAM), while TAM enhances the transcription of M2-like genes *hypoxia-inducible factor 2α (HIF-2α), ARG1,* and *VEGF*.^20,206,283–285^ This polarization-inducing function may be mediated through extracellular signal-regulated kinase (ERK)/signal transducer and activator of transcription 3 (STAT3) pathway.^286^ Crosstalk with other epigenetic regulatory mechanisms may also occur during the lactate-induced M2 polarization. As a carbon source for the TCA cycle, lactate induces adenosine triphosphate–citrate lyase (ACLY)-dependent histone H3 lysine 9 acetylation (H3K9ac) in BMDM, thereby upregulating the expression of M2-like genes.^287^ Besides, lactate promotes tumor progression by reprogramming phenotype of microglia and monocyte/macrophages. Lactate is a pro-inflammatory mediator which encourages interleukin-23 (IL-23) transcription in Toll-like receptor (TLR)-stimulated monocytes and macrophages, thereby sustaining IL-23-dependent interleukin-17 (IL-17) secretion and polarizing the immune response against T_H_17 cells.^288^ Downregulation of LDHB skews TAMs to function as a lactate and sterol/oxysterol source for the proliferation of breast tumor cells.^289^ Additionally, as sentinel cells in the central nervous system, microglia upregulate the expression of insulin-like growth factor-binding protein 6 (IGFBP6) in response to lactate, thereby promoting M2 polarization and recruitment of microglia in the zebrafish GBM model.^290–292^ Then, H3K18la of TAM prohibited expression of retinoic acid receptor γ (RARγ), elevated IL-6 levels in the TME and activated STAT3 signaling in CRC cells, which in turn empowered macrophages to promote tumorigenesis.^293,294^

When it comes to Treg cells, the lactate-rich environment which is rich in lactate permits proliferation and immunosuppressive effect of Treg cells.^281,295,296^ Raychaudhuri et al. found that lactate weakened interferon-α (IFNα) induction and enhanced the recruitment of FoxP3 + CD4+ regulatory T (Treg) cells by plasmacytoid dendritic cells (pDCs) in a mouse breast cancer model. This impairment boosted the expansion of a specific group of Treg cells and promoted an immunosuppressive TME.^297^ Moreover, lactate fosters programmed death-ligand 1 (PD-1) level in Treg cells in rich-glycolysis TME, giving rise to treatment failure of immunotherapy.^298,299^ As the tumor-infiltrating Tregs require lactate uptake to sustain their immunosuppressive function, Treg-specific deletion of MCT1 demonstrates that while lactate blockage is not necessary for the functioning of peripheral Treg cells, it is essential within TME and leads to an impaired tumor growth and enhanced sensitivity to immunotherapy.^300,301^ To add up, Chen et al. manifested that lactate facilitates the lactylation of APOC2 at K-70, which stabilizes the protein and subsequently leads to the accumulation of Treg cells, immunotherapy resistance, and tumor metastasis of NSCLC.^181^

Furthermore, with respect to T cells and natural killer (NK) cells, Daneshmandi et al. observed elevated infiltration of NK cells and CD8+ cytotoxic T cells in melanoma cells deficient in LDHA.^302^ Additionally, Liu et al. found that lactate produced by KRAS-mutant colorectal cancer cells diminishes the sensitivity to anti-PD-1 therapy by inactivating nuclear factor-kappa B (NF-κB) and sensitizing CD8 + T cells to activation-induced cell death (AICD).^303^ Furthermore, during this process, circular RNA CircATXN7 may play a crucial role in inducing NF-κB inactivation and regulating T cell sensitivity to AICD. It has been identified as a potential target for enhancing anti-PD-1 therapy in mouse models of colorectal cancer, pancreatic cancer, and melanoma.^304^ Apart from that, Chang et al. demonstrated that prostate cancer released 1-Pyrroline-5-carboxylate (P5C) which inhibited T cell glycolysis through enhancing the activity of LDHB.^305^ With regard to mechanisms, lactate hinders IFN-γ production by downregulating T cell receptor (TCR)-triggered phosphorylation of JNK, c-Jun, and p38 in Cytotoxic T lymphocyte (CTL).^306^ Research also revealed that tumor-produced lactate inhibited focal adhesion kinase interacting protein (FIP) expression by downregulating nicotinamide adenine dinucleotide levels and simultaneously sensitizing the inhibitory effect of the adenylglycine uridylic acid-rich element in the untranslated region of the Fip200 mRNA, targeting naïve T-cells to evade the immune response.^307^ Besides, lactate and H+ ions exported to the TME impair immune surveillance of effector T cells and NK cells by respectively inhibiting glycolytic flux, granzyme B and IFN-γ secretion.^23,302,308^

More to the point, lactate-modulated immunosuppression hinders treatment sensitivity.^309–313^ Lin et al. uncovered that radiotherapy enhanced glycolysis and lactate secretion in pancreatic cancer, which bolstered myeloid-derived suppressor immune cells (MDSCs) and promoted a suppressive immune microenvironment, which in turn led to pancreatic cancer progression and recurrence.^314^ In brief, lactate modulates immune cells in TME, resulting in impaired differentiation, decreased immune response, evasion of immune surveillance, and treatment resistance.

Nonetheless, the latest research has revealed divergent viewpoints. Lactate stimulates the production of antitumor cytokines, such as IFNγ, IL-2, and TNFα, in T cells and boosts the proliferative and cytotoxic capabilities of CD8 + T cells.^315^ Furthermore, administering sodium lactate intraperitoneally (2 g/kg) results in reduced subcutaneous tumor growth of breast cancer, cutaneous melanoma, LLC and colon adenocarcinoma, with the effect being dependent on T cells.^315^ Besides, it is reported that lactate hinders the differentiation and promotes stemness of T cells, thus enhancing anti-tumor immunity. Feng et al. recently discovered that high sodium lactate concentrations boosted histone H3 lysine 27 acetylation (H3K27ac) levels at the T cell factor 7 (TCF7) super-enhancer locus by inhibiting histone deacetylase activity. This, in turn, led to higher T cell factor 1 (TCF1) expression and enhanced the stemness of CD8 + T cells.^316–318^ Lactate-induced extracellular acidosis blocks one-carbon metabolism that short-lived effector T cells are highly dependent on, subsequently bringing about decreased histone 3 lysine 27 trimethylation (H3K27me3) deposition of memory-related genes, thereby affecting differentiation, reserving sternness and facilitating anti-tumor cytotoxicity of T cells.^318–320^ It implies that lactate metabolism reprogramming towards T cells may be a double-edged sword which elicits a sophisticated effect on its antitumor immunity, which waits to be further investigated.

### Stromal cells

Cancer-associated fibroblasts (CAFs) are essential components of the TME with multiple roles, encompassing stromal remodeling and deposition, extensive bidirectional signaling interactions with cancer cells, and communication with immune cells.^321–324^ Basal membrane (BM) remodeling is the process by which cells regulate cell-BM interactions by changing the structure and composition of BM.^325–327^ EMT describes the transformation from epithelial cells to mesenchymal cells.^328–331^ CAFs synthesize type I collagen and intensify tumor invasiveness by promoting BM remodeling and EMT.^332,333^ In CAFs coupled with prostate carcinoma cells models, contact between tumors and the stroma-activated CAFs through stabilization of HIF-1 that rely on sirtuin 3 or succinate, initiating mitochondrial oxidative stress, promoting mitophagy, upregulating expression of GLUT1 and glycolytic enzymes, and facilitating lactate biosynthesis. Meanwhile, CAFs metabolically reprogramed tumor cells so that prostate carcinoma cells tended to metabolize CAFs-sourced lactate rather than glucose through glycolysis.^321^ In parallel to promoting malignant phenotypes by facilitating BM remodeling, EMT, and metabolic reprogramming, CAFs have also been associated with treatment resistance. Apicella et al. revealed that the metabolic shift in tumor cells induced by tyrosine kinase inhibitors (TKIs) targeting mesenchymal-epithelial transition factor or epidermal growth factor receptor (EGFR), which results in increased lactate production, prompts CAFs to excessively produce HGF. This process ultimately reinforces drug resistance and promotes tumor progression.^334^

### Endothelial cells

In addition to enhancing tumor invasion and metastasis via tumor cell lactate autocrine as mentioned above, paracrine secretion of lactate can directly modulate endothelial cell phenotype, thereby altering tumor vascular morphogenesis and perfusion.^335–338^ As for non-malignant endothelial cells at regular oxygen concentrations, lactate activates hypoxia-inducible factor 1α (HIF-1α) that enhance*s basic fibroblast growth factor (bFGF) and vascular endothelial growth factor receptor 2 (VEGFR2)* expression, which synergizes with lactate-induced VEGF secretion.^339^ Lactate from tumor cells and stromal cells can enter endothelial cells via MCT1, promoting 2-oxoglutarate-dependent prolyl hydroxylase (PHD2) and ROS-dependent NF-κB activation in endothelial cells.^340^ Subsequently, endothelial cells produce IL-8, which mediates angiogenesis via autocrine.^135^ In addition to triggering the MCT1/NF-κB/IL-8 pathway, it was also found that lactate promotes angiogenesis by stimulating the phosphoinositide 3-kinase/protein kinase B signaling (PI3K/Akt) pathway. This activation developed through the engagement of three receptor tyrosine kinases—AXL receptor tyrosine kinase (Axl), TEK receptor tyrosine kinase 2 (Tie2), and VEGFR2 in endothelial cells.^341^ A-cyano-4-hydroxy-cinnamate (CHC) is an inhibitor of MCT1, which inhibits lactate metabolism.^342–344^ MCT1 inhibition downregulates *VEGF* expression, blocking lactate-induced endothelial cell migration, vascular outgrowth, and human umbilical vein endothelial cell (HUVEC) tube formation. The results of mouse experiments were consistent with cytological experiments that subcutaneous lactate matrix plugs promoted angiogenesis and that inhibitors of MCT1 inhibited angiogenesis in mouse HCC subcutaneous tumor model.^339^

## Clinical application and interventions

Disruption of lactate homeostasis is one of the major mechanisms of tumor-targeted therapy.^345–347^ The major targets for lactate production and transport are LDH and MCTs^348^ (Table 2). LDH transforms pyruvate to lactate, and inhibition of its activity reduces lactate production. Besides, MCTs promote lactate transport. Targeting them disrupts lactate from release. On top of that, altered lactate levels may result in a therapeutic effect single-handedly or synergize with other conventional adjunctive anti-tumor treatments such as immunotherapy, chemotherapy, and thermotherapy for sensitizing effects.

**Table 2:** Onco-therapeutic drugs targeting lactate metabolism/lactylation

Target	Drug name	Reserch status	Applications	Mechanism	Ref/Trial No.
LDHA	AT-101(Gossypol) and its derivative e.g., FX-11	Preclinical	Pancreatic cancer, breast cancer, adrenocortical carcinoma, and lymphoma	Lactate anabolism	^26,34,188,382,383,498^
LDHA	oxamate	Preclinical	NSCLC, melanoma	Lactate anabolism	^190,389,391^
LDHA	GSK2837808A	Preclinical	Melanoma	Lactate anabolism	^392^
LDHA	GNE-140	Preclinical	Pancreatic cancer	Lactate anabolism	^385^
LDHA	Zinc	Preclinical	Melanoma	Lactate anabolism	^394^
LDHA	NHI-Glc-2	Preclinical	NSCLC and gastric cancer	Lactate anabolism	^334^
LDHA	LncRNA GLTC	Preclinical	PTC	Lactate anabolism	^393^
LDHA/B	NCI-006	Preclinical	Pancreatic cancer	Lactate anabolism	^386^
LDHA/B	MS6105	Preclinical	Pancreatic cancer	Lactate anabolism	^387^
MCT	CHC	Preclinical	Breast cancers	Lactate anabolism	^342–344^
MCT1/2	AR-C155858 (SR13801)	Preclinical	Breast cancers, B-cell lymphoma	Lactate anabolism	^397,398,499^
MCT1/2	AZD3965	Clinical trial	Advanced solid cancers (NSCLC, SCLC, prostate, gastric, breast cancers), Burkitt’s lymphoma and diffuse large B-cell lymphoma	Lactate excretion	NCT01791595^76,79,178,334,344,398,496^
MCT1	AZ3965	Preclinical	CRC, melanoma	Lactate excretion	^400^
MCT1	AR-C122982 (SR13800)	Preclinical	Burkitt lymphoma cell, neuroblastoma	Lactate excretion	^399,500^
MCT1	BAY8002	Preclinical	HCC, some solid cancers	Lactate excretion	^400^
MCT4	AZ93	Preclinical	CRC, melanoma	Lactate excretion	^410^
MCT4	ALK-04	Preclinical	Melanoma	Lactate excretion	^492^
MCT1/4	Syrosingopine	Preclinical	HCC, lung cancer, hematologic malignancies, breast cancer	Lactate excretion	^166,501,502^
MCT1/4	7ACCs	Preclinical	Cervix cancer, breast cancer, CRC	Lactate excretion	^102,503^
CD147	AC-73	Preclinical	HCC, AML	CD147-MCT interaction	^413,504^
CD147	metuzumab	Preclinical	LUAD, NSCLC	CD147-MCT interaction	^414^
Lactate	LOx	Preclinical	Breast cancer, melanoma, GBM, HCC	Lactate catabolism	^418–425,428–431,495^
Lactate	NaHCO_3_	Preclinical	NSCLC, breast cancer	Lactate catabolism	^434,494^
Lactate	nanoCaCO_3_	Preclinical	Βreast cancer	Lactate catabolism	^435^
Lactate	CoMnFe-LDO	Preclinical	UM	Lactate catabolism	^493^
GLUT1	BAY876	Preclinical	Breast cancer, GBM	Lactate anabolism	^432,505^
Pyruvate	UK5099. POx	Preclinical	Breast cancer	Pyruvate catabolism	^433^
Lactylation	BML	Preclinical	HCC	Histone modification	^225^
Lactylation	RJA	Preclinical	HCC	Histone modification	^226^
Lactylation	K673-pe	Preclinical	CRC	Non-histone modification	^243^
Lactylation	D34-919	Preclinical	GBM	Non-histone modification	^244,436^
Lactylation	anti-APOC2^K70-lac^ antibody	Preclinical	NSCLC	Non-histone modification	^181^
Lactylation	β-alanine	Preclinical	CRC	Non-histone modification	^239^
Lactylation	MG149	Preclinical	CRC	Non-histone modification	^240^

*AML* acute myeloid leukemia, *CD147* cluster of differentiation 147, *CRC* colorectal cancer, *GBM* glioblastoma, *GLUT1* glucose transporter 1, *HCC* hepatocellular carcinoma, *LDHA* lactate dehydrogenase A, *LUAD* lung adenocarcinoma, *MCT* monocarboxylate transporters, *MCT1* monocarboxylate transporter 1, *MCT2* monocarboxylate transporter 2, *MCT4* monocarboxylate transporter 4, *NSCLC* non-small cell lung cancer, *SCLC* small cell lung cancer, *UM* uveal melanoma

### Novel detection methods for lactate

Advanced molecular and imaging techniques are providing new insights into the mechanisms and functional importance of the fluctuation in tissue lactate/lactylation levels that occur during tumor progression.^349–351^

As for chemical probe for lactate detection, a fluorescently tagged analogue of L-lactate was employed as an L-lactate mimic to explore its transportation and metabolic processing within live cells.^352^ Aside from lactate-detecting probes, single-cell technique-based metabolomics analysis introduces a computational framework for profiling lactate metabolism and outlines key principles of the TME.^353–357^ For instance, metabolite set enrichment analysis revealed that metabolic pathways associated with the Warburg effect are linked to the metastatic potential of CRC cell lines.^358^ Subsequently, using a custom-built single-cell quantitative mass spectrometry platform, researchers monitored 14 identified metabolites in individual circulating tumor cells from CRC patients and developed a 4-metabolite fingerprint classifier, which includes lactate, to efficiently predict metastasis risk.^359^ Moreover, using isotope-tracing-based metabolic flux analysis, researchers can trace the path of each isotopic carbon atom, thereby assisting in gaining deeper insights into the particular intermediates and detailed metabolic processes caused by lactate.^318^ For example, with the assistance of isotope-tracing analysis, researchers proved that ^13^C-glucose labeled TCA intermediates were superior to that with ^13^C-lactate label in the brain, TCA labeling from lactate was significantly higher than infused ^13^C-glucose in other tissues.^143^ Besides, using ^13^C-labeled metabolic flux assays, researchers found that the preference for glycolysis and OXPHOS varies across variant stages of the cell cycle in breast cancer cell lines. Cells in the G1 phase primarily prefer OXPHOS, while cells in the S phase predominantly prefer glycolysis.^360,361^ To add up, by intravenously administering primed [U-^13^C]lactate (completely labeled with carbon-13 at three positions) and utilizing imaging mass spectrometry (IMS), Bartman et al. mapped TCA cycle flux across various tumor models.^14,362,363^ Their findings revealed that TCA flux in primary solid tumors, such as pancreatic cancer, NSCLC, and CRC, was lower than in corresponding normal tissues, while in hematological malignancies like NOTCH1-driven T cell acute lymphocytic leukemia, TCA flux in the spleen was elevated compared to normal spleen tissue.^39,364,365^ Additionally, in a breast cancer lung metastasis model, metastatic sites exhibited higher TCA flux compared to primary sites. These results suggest that despite an increase in glycolytic flux in tumors relative to normal tissues, the rate of ATP production is reduced.^366,367^ In addition, as for metabolic imaging, Li et al. reported a high-performance imaging technique for monitoring lactate, named FiLa, which achieves in situ, real-time, and quantitative dynamic tracking of lactate metabolism in live cells, subcellular structures, and in vivo.^368^ This method has made significant advances in understanding lactate spatial distribution, regulatory networks, drug screening, and clinical diagnostics. The FiLa probe, used for detecting lactate levels in subcellular organelles, showed that lactate concentrations in the nucleus are comparable to those in the cytoplasm, while mitochondrial lactate levels are markedly higher than those in the cytoplasm and nucleus.^369^

Since lactylation is a relatively new discovery, its presence on non-histone proteins and its subsequent functional impacts are not yet well unearthed, which proposes urgentexpectations for accurate detection methods of lactylation. Wan et al. presented a cyclic immonium (CycIm) ion of lactyllysine (Klac) that formed during tandem mass spectrometry, which allowed for precise assignment of protein lactylation. The sensitivity and specificity of this lactylation detecting method were confirmed through affinity-enriched lactylproteome analysis and extensive informatic evaluation of non-lactylated spectral libraries.^370^ Sun et al. also invented a chemical lactylation probe named sodium (S)-2-hydroxypent-4-ynoate (YnLac) which metabolically integrated into lactylated proteins, allowing them to be directly tagged with fluorescent or affinity markers for fluorescence visualization or proteomic analysis.^371^ Moreover, single-cell technologies play a crucial supportive role in studies related to tumor lactylation. It enables detailed correlation analyses based on cell type and the lactylation level, revealing genes associated with lactylation of specific cell population. It provides multi-dimensional indicators for evaluating tumor metabolic phenotypes and predicting tumor prognosis and has been applied in CRC.^372,373^ In summary, advancements in detection technologies are pressingly required to offer fresh insights into whether and how lactate/lactylation influences a plethora range of biological processes in different cell populations and plays a role in oncology.

### Targeting LDHs

LDH is a tetrameric enzyme that mediates bidirectional transformation between pyruvate and lactate. LDHA is the prevailing isoform utilized by cancer cells to bypass OXPHOS. This diverts metabolic precursors of pyruvate into the pentose phosphate pathway, which supports cancer cell proliferation.^74,189,374,375^ A high level of LDHA indicates poor prognosis in several human malignancies.^189,376,377^ Meanwhile, overexpression of LDHB has been found in plenty of different cancers, including breast, thyroid, lung, and pancreatic cancer, which is significantly associated with unfavorable prognosis.^378–380^ Current efforts are focused on development of LDH inhibitors with better cellular potency, PK properties, and selective compounds and remain in preclinical state. AT-101 (gossypol), as an EGFR mutation targeted therapy, has been used in phase I/II randomized clinical trials of advanced non-small cell radiation-induced lung cancer, head and neck cancer and metastatic castration-resistant prostate cancer. Thus, standard chemotherapy with AT-101 has achieved potential benefits in high-risk patients or some patients with prolonged progression-free survival or overall survival (NCT01003769, NCT00988169, NCT00286780, NCT00540722).^381^ Nonetheless, AT-101 and its derivative FX-11, galloflavin, and N-hydroxyindole-based compounds are promising cell-active LDHA inhibitors, which pharmaceutic effect hasn’t been applied in clinical practice.^26,382,383^ Intravenous injection of LDHA/B inhibitor NCI-006 inhibits LDH activity and its growth in pancreatic cancer mouse models, so as oral administration of LDHA inhibitor GNE-140.^384–386^ Moreover, LDH PROteolysis TArgeting Chimeras (PROTAC) degrader, MS6105, time and ubiquitin-proteasome system-dependently degrades LDHA/B and inhibits the proliferation in multiple pancreatic cancer cell lines.^387^ The efficacy of LDHA inhibitors is limited due to LDH between different tumors and metabolic reprogramming-mediated LDH isoform transformation.^343,388^ Furthermore, a combination of LDHA inhibitor oxamate and respiratory complex I inhibitor metformin retards tumor progression in melanoma mice models.^389^

LDH-targeted therapy also achieves curative effect when combined with adjuvant treatments. Regardless of its effect as a tumor suppressor, emerging evidence validates the role of LDH inhibitors as an immunotherapy sensitizer.^390^ Using oxamate and PD-1 blockade pembrolizumab stimulated CD8 + T cell infiltration and hindered tumor proliferation in humanized mouse NSCLC model, thereby sensitizing immunotherapy.^391^ In preclinical melanoma mouse model, LDHA inhibitor GSK2837808A increased therapeutic effect of adoptive T cell therapy (ACT).^392^ Likewise, LDH inhibitors contribute to photothermal therapy (PTT). Zhao et al. Constructed a Zinc-enriched nanosystem which contained both glycolysis inhibitor LND and LDHA inhibitor Zinc for combined glycolysis modulation and photothermal therapy. In addition, LDHA inhibition induced by oxamate led to the accumulation of ROS and depletion of cellular ATP, leading to DNA damage, DNA repair activity impairment and boosted radiotherapy efficiency in NSCLC.^190^ Moreover, LDHA inhibitor restores sensitivity towards radioiodine (RAI) in papillary thyroid cancer (PTC). Shi unvealed that long noncoding RNAs (lncRNAs) glycine-rich long non-coding transcript (GLTC) hindered the succinylation of LDHA at K-155 by impeding the competitive inhibition of GLTC against the binding of sirtuin 5 (SIRT5) to LDHA. This restraint of LDHA enzymatic activity inhibited tumor progression and resistance to RAI in PTC.^393^

Results demonstrated that the presence of free zinc ions led to a concentration-dependent inhibition of LDHA activity and an elevation in LDH efflux, invigorating PTT treatment and synergistically suppressed primary melanoma and lung metastasis.^394^

### Targeting MCTs

Targeting MCTs exerts significant effects on metabolic symbiosis.^132^ There are a variety of MCT inhibitors, including CHC,^342–344,395^ organomercurial compounds,^396^ photothialdehyde benzenesulfonate,^396^ as well as second-generation pharmaceuticals of more acceptable selectivity, such as AR-C155858 for MCT1/2^397,398^ and BAY8002, SR13800 for MCT1.^399,400^ In addition, AstraZenec’s compound AZ3965, which targets MCT1/2, has shown promising results in preclinical studies in small cell lung cancer (SCLC).^78^ Surely, AZ3965 is also therapeutically effective in models of MCT1-positive Burkit’s lymphoma, breast and gastric cancers.^78,399^ AZD3965 has already finished a Phase I/II clinical trial (NCT01791595) in patients with solid tumors diffuse large B-cell lymphoma,^76,79^ which reveals its pharmacokinetic characteristics and adverse effects and suggests that AZD3965 is tolerated at doses that produce target engagement. Dose-limiting toxicities were on-target and primarily dose-dependent, asymptomatic, reversible ocular changes. Preclinical evidence and retrospective analyses suggest MCT4 may serve as a compensatory option for MCT1 activity as long as MCT1 is downregulated. This study suggests the complexity of targeting MCT and potential resistance mechanisms.^78^ Moreover, targeting MCT1 boosts tumor reactivity of CD8 + T cells by exerting influence on lactate catabolism.

In addition to MCT1 inhibitors, MCT4 inhibitors have shown promising applications. In hypoxic TME, MCT4 expression is induced by HIF1α.^38,104^ Knocking down MCT4 reverses the changes in sensitivity of lung adenocarcinoma cell lines to glycolysis inhibitors and OXPHOS inhibitors under hypoxic conditions, indicating the vital role of MCT4 in lactate-targeted therapy.^401^ MCT4i is a promising therapeutic choice for gastric cancer,^402^ colorectal cancer,^403^ breast cancer,^404,405^ prostate cancer,^406^ lung adenocarcinoma (LUAD),^407^ and GBM.^408,409^ The combination of MCT1i (AZ3965) and MCT4i (AZ93) significantly inhibited proliferation in colorectal cancer cell lines.^410^ 7-Aminocarboxycoumarins (7ACCs) compounds prevented MCT1 compensation resulting from MCT4 inhibition by simultaneously suppressing both MCT1 and MCT4, down-regulating mitochondrial pyruvate transport leading to intracellular pyruvate accumulation, and blocking lactate inward compensation.^102^ The assistance of the chaperone molecular chaperones CD147 or basigin (BSG) assures expression of MCT1 and MCT4 at the plasma membrane.^411,412^ Preclinical models of prostate cancer show that inhibition of CD147/BSG achieves modulation of lactate transport through MCT1/MCT4 activity, reducing lactate efflux and tumor growth.^397^ The CD147 dimerization inhibitor AC-73,^413^ the human/mouse chimeric IgG1 mAb of CD147 named metuzumab,^414^ the organomercurial reagent p-chloromercuribenzene sulfonate (pCMBS),^415^ which blocks MCT1/MCT4-CD147 binding 118 are available CD147-targeted anticancer drugs. Nonetheless, on a cautionary note, CD147 is ubiquitously expressed and interacts with other proteins at the cell surface. Thus, strategies selectively targeting CD147-MCT interactions ought to minimize drug toxicity and establish a therapeutic window.^26^

There is evidence that integration of MCT-targeted and other therapies fulfill a better therapeutic role. Li et al. revealed that in breast cancer mouse model, MCT inhibitor Syrosingopine downregulated the number of Treg cell and upregulated that of NK cells and M1 phenotype of TAM, suggesting reversal of the immunosuppressive TME.^416^ Meanwhile, Ma et al. found that in vivo and in vitro, Lithium carbonate (LC) assisted MCT1 localization to mitochondrial membranes and lactate influx into mitochondria. Revitalization of tumor-reactive CD8 + T cells induced by above-mentioned extra energy support sensitized immunotherapy towards CRC, melanoma and breast cancer.^417^ It sheds insight on the aspect that MCT targeted therapy may fulfill a role in synergy with immunotherapy.

### Other lactate-targeted strategies

While development of LDH and MCT inhibitors is in full swing, there is emerging concern that they can disrupt the metabolism of healthy cells and cause severe non-specific toxicity. As for solution to overcoming these shortcomings, researchers put forward the opinion that lactate oxidase (LOx) was a therapeutic option which reduced lactate concentrations, released H_2_O_2_ and recruited immune cells, overcoming immunosuppression and sensitizing immunotherapy.^418,419^ Moreover, the drug delivery system of LOx evolves gradually from polymer nanocarriers into self‐assembled nanoparticles, the update refinement of which empowers its application towards chemotherapy and sonodynamic therapy (SDT) sensitization.^420–424^ The depletion of lactate catalyzed by LOx generates pyruvate, which in turn activates clustered regularly interspaced short palindromic repeat-associated protein 9 (CRISPR/Cas9)-mediated signal-regulatory protein alpha (SIRPα) genome-editing plasmids. When combined with a metal-organic framework (MOF), LOx and these plasmids are utilized to form nanoparticle named LPZ (LOx, Cas9/sgSIRPα plasmids, mannose-modified PEG loaded-ZIF-67) and facilitate the conversion of M2 macrophages to M1 macrophages, thereby inhibiting the growth of in situ breast cancer models.^425^ This approach offers a method for LOx-induced-CRISPR/Cas9-mediated macrophage gene editing directly within the tumor site and presents a potential strategy for enhancing immunotherapy.^426,427^ For instance, Luo et al. utilized nano-ZIF-8 as the carrier to construct the Hb-LOx-DOX-ZIF8@platelet membrane nanosystem (HLDZ@PM NPs) and effectively enhance the tumor sensitivity to DOX-induced chemotherapy.^428^ Anchor LOx onto the surface of lactobacillus (LA) also increased lesion targeting and delivery efficiency, enabled LOx to fully catalyze lactate oxidation and depletion of intra-tumor oxygen, thus activating the chemotoxicity drug to induce apoptosis.^429,430^ Zhang et al. developed a metal-phenolic network-based nanocomplex, incorporating LOx and the mitochondrial respiration inhibitor atovaquone (ATO) to reconstruct the immunosuppressive TME. This nanocomplex demonstrated superior pharmacological effects compared to single-agent therapy in breast cancer SDT.^431^

Besides, the newly-reported targeting strategy also aims to block AARS1, which serves as a bridge linking tumor cell metabolism with proteomic changes. Β-Alanine blocked the interaction between AARS1 and lactate, preventing subsequent lactyl transfer. As a result, the tumor suppressor gene p53 was not lactylated at K120 and K139, which inhibited tumor progression in a CRC mouse model.^239^ Downregulation of pan-Kla writer KAT8 by the histone deacetylase (HDAC) inhibitor MG149 blocks the KAT8-eEF1A2 Kla axis and suppresses CRC tumor growth, particularly in a high-lactic TME.^240^

Likewise, there are other elucidated strategies of dual regulation of metabolism and immunity. Li et al. invented an in-situ injection of a thermogel loaded with glucose transporter 1 (GLUT1) inhibitor-sensitized GBM immunotherapy of PD-1/PD-L1 blocker BMS-1 through alleviating lactate-driven Treg cells.^432^ Additionally, Niu et al. invented a novel single-atom nanozyme pyroptosis initiator: UK5099 and pyruvate oxidase (POx)-co-loaded Cu-NS single-atom nanozyme (Cu-NS@UK@POx), offering a dual-pronged approach that effectively enhanced the immunotherapeutic anti-tumor effects by inducing ROS storms and lactate/ATP depletion.^433^ Aside from the oxidative catabolism of lactate, neutralizing it with a basic salt is another potential strategy for targeted therapy. Inhibiting spontaneous metastases in mouse models of metastatic breast cancer was shown to be effective by neutralizing lactate in the tumor using a basic salt such as NaHCO_3_.^434^ Besides, acid-neutralizing CaCO3 nanoparticles were used to maintain the pH within the normal physiological range in breast cancer cells, which inhibited the proliferation and migration.^435^ To sum up, as other intervention in tumor lactate metabolism shows great potential to of chemotherapy and immunotherapy sensitization. The optimal scheme of organically combining lactate metabolic regulation with other therapies, which includes screening the most effective lactate regulation target, determining the best treatment time window, and identifying the most appropriate action site to maximize antitumor efficacy, remains to be further explored. Further exploration is needed to determine the optimal approach for effectively integrating lactate metabolic regulation with other therapies. This includes identifying efficient target, determining the optimal treatment time window, and pinpointing the most suitable action site to maximize the anti-tumor efficacy.

By inhibiting H3 histone lactylation (H3K9la and H3K56la), demethylzeylasteral decreased the tumorigenesis driven by liver cancer stem cells (LCSCs) both in vivo and in vitro. This indicated that lactylation inhibition served as a potential candidate for adjunctive tumor therapy.^225^ Besides, Xu et al. also found that by downregulating lactylation at H3K9la and H3K14la, lactate production was reduced by royal jelly acid (RJA), thereby inhibiting tumor invasion, migration, proliferation, and apoptosis of HCC.^226^

Inhibition of non-histone lactylation at the MRE11 K673 site through K673-peptide-3# (K673-pe) suppressed HR in CRC, thereby restoring its sensitivity to chemotherapy and poly ADP-ribose polymerase inhibitor (PARPi). Hearin, K673-pe exhibited a synergistic tumor-suppressive effect when combined with chemotherapy.^243^ D34-919 blocked the interaction between ALDH1A3 and PKM2 in GBM cells, thereby suppressing the downstream lactylation of XRCC1, which restored the sensitivity of GBM to TMZ-based chemotherapy and radiotherapy in GBM organoid models.^244,436^

Potential targeted therapeutic approaches may be possible by targeting lactate production and transport, particularly through LDH and MCTs. However, because these molecules are involved in complex interactions that control multiple signaling and metabolic events, it is challenging to determine the quantitative contribution of modulated lactate balance to therapeutic efficacy.^437,438^ In addition, metabolic targeting depends on specific metabolic requirements, by way of example, lactate metabolism-targeting drugs are ineffective against glutamine-dependent cells. The metabolic heterogeneity of diverse tumor types, varied clinical stages, distinct cell populations within the TME may be one of the factors contributing to the poor efficacy of metabolic-targeted drugs.^439–441^ To elaborate, in the study by Liu et al., it was shown that when the metabolic pathway of HCC, the most common type of primary liver cancer, shifted from glycolysis to OXPHOS, the proliferation of HCC cells and tumor growth were inhibited.^442^ However, for cholangiocarcinoma (CCA), the second most common type of primary liver cancer, mitochondrial OXPHOS metabolism helps maintain stem-like characteristics in CCA, thereby conferring tumorigenic potential and chemotherapy resistance in vivo.^158,443^ Apart from primary liver cancer, PD-1 resistant NSCLC cells were characterized by a markedly lower glycolytic reserve and exhibited a significantly higher reliance on OXPHOS compared to PD-1 sensitive NSCLC cells, which implies that tumor progression leads to complex changes in lactate metabolism flux.^159,441^ As well as primary liver cancer and NSCLC, CRC exhibits different lactate metabolism across various histological and molecular subtypes.^444,445^ Based on global genomic profiling, CRC can be classified into microsatellite stable (MSS) CRC and microsatellite unstable (MSI) CRC, with differences observed in mitochondrial DNA (mtDNA) copy numbers between these two types.^446^ Sun et al. found that MSS cancer cells with higher mtDNA copy numbers tended to rely on OXPHOS, which promoted malignant tumor development.^447^ Conversely, MSI cancer cells with lower mtDNA copy numbers depended on glycolysis, which contributed to tumorigenesis and cisplatin chemoresistance.^160,446,448^ In various cancers such as gastric cancer, breast cancer, HCC, NSCLC, endometrial cancers (EC) and renal cell carcinoma (RCC), mtDNA levels are decreased.^449–451^ Conversely, mtDNA copy numbers are elevated in other cancer types, including CRC, esophageal squamous cell carcinoma, acute lymphoblastic leukemia, head and HNSCC, and ovarian cancer.^452–457^ This suggests both complexity and potential commonalities in lactate metabolism characteristics across tumor types.

Meanwhile, when applied to tumors characterized with unlocking phenotype plasticity, lactate inhibitors are characterized with a high off-target rate and low specificity to lactate metabolism, which obscures their true pharmacological mechanisms and therapeutic effects.^383,384,458,459^ In this situation, targeting the metabolic symbiosis between cancer and stromal cells by inducing metabolic switching in the tumor TME leads to sensitization of targeted therapies. For instance, anti-angiogenic drugs may elicit a more hypoxic TME, thereby reducing the metabolic heterogeneity of the TME and enhancing the efficacy of targeted therapies. However, metabolic malleability in invasive tumors results in recurrence as well as drug-resistant clones with more complex metabolic profiles. Last but not least, changes in the non-cancerous cells during treatment are another being issue that metabolic targeting may confront. Lactate not only provides energy to neurons but also spreads beyond the active zone, influencing the function of neurons and astrocytes in nearby regions.^179^ Playing a role in processes ranging from neurovascular coupling to learning and memory, lactate serves a dual purpose as both a metabolic fuel and an intercellular signaling molecule.^460,461^ On the one hand, in relation to normal cells which lactate/lactylation exerts a pivotal effect on its physiological functions, lactate/lactylation targeted therapy may inflict a fatal blow to their structure and operation.^462–464^ For example, playing a role in processes ranging from neurovascular coupling to learning and memory, lactate serves a dual purpose as both a metabolic fuel and an intercellular signaling molecule.^91,349^ Also, current research demonstrated the critical role of lactate and lactate transport enzymes, such as LDHA, in biogenesis and function of phototransduction system.^465–468^ Lactate stimulated by insulin from the retinal pigment epithelium (RPE), supports the crucial visual functions of photoreceptors by enhancing glucose absorption in the retina.^465^ Last but not least, notably, the interplay between epigenetic mechanisms complicates the selective targeting of histone lactylation without influencing acetylation.^21,469,470^ On the other hand, immune system activation is accompanied by changes in immune cell metabolism.^471^ As an illustration, the activation of lymphocytes during the anti-tumor process of the immune system is dependent on the Warburg effect. Moreover, lactate, through arrestin β-2 (ARRB2) and GPR81, inhibits Toll-like receptor (TLR)-induced activation of the NOD-like receptor family pyrin domain containing 3 (NLRP3) inflammasome and the production of interleukin-1 beta (IL-1β).^180^ Thus, if an antimetabolic compound impairs tumor cell growth, it can also inhibit the anti-tumor immune response and anti-inflammasome-mediated inflammation immunomodulation.^472,473^

To overcome the heterogeneity and adaptability of tumor metabolism, enhance the specificity of targeted drugs, minimize adverse effects, and hinder the occrurence of treatment resistance, novel investigations in targeted therapy focus on devising novel small-molecule inhibitors, developing robust delivery systems that adhere to the 3 R delivery principle (right site, right timing and right dose) and exploring rational and effective drug combination strategies.^474–476^

Regarding novel lactate/lactylation inhibitors, PROTAC are novel small-molecule inhibitors that downregulate or even eliminate their target proteins via the ubiquitin-proteasome pathway.^477^ As PROTAC effectively degrade target proteins even under conditions of low affinity, they avoid several drawbacks associated with traditional small-molecule inhibitors, such as the need for high specificity to protein binding pockets, the development of resistance due to mutations at binding sites after prolonged use, and the cumulative toxicity from sustained high concentrations.^478–480^ Furthermore, PROTAC have successfully achieved dual targeting of LDHA/LDHB and proliferation inhibition in pancreatic cancer cell lines. Following intraperitoneal injection, the plasma of mice also indicated favorable drug bioavailability.^387^ This suggests that PROTAC could serve as a useful molecular tool for in-depth research to target lactate metabolism.

Considering delivery systems to effectively restore aberrant lactate and lactylation levels, catalytically active nanomaterials and gene-editing techniques have shown the therapeutic potential in previous studies.^468,481^ Catalytically active nanomaterials, often known as “nanozymes,” have emerged as promising alternatives. They present numerous benefits, such as improved catalytic efficiency, resistance to harsh environments, prolonged effectiveness, and precise targeting.^421,482,483^ These benefits grant nanozymes diverse and potent therapeutic potential in delivering small molecule lactate/lactylation inhibitors.^479^ Apart from nanozymes, gene editing techniques have also demonstrated possible functions in steamlinedly regulating lactate and lactylation. Adeno-associated virus (AAV) is viewed as among the leading viral vectors for gene delivery due to its capacity to infect diverse tissue types and its reputation as a comparatively low-risk option for gene transfer.^484–488^ AAV8-packaged vectors have been utilized for sclera-specific gene editing, which was employed to investigate how enhanced lactate/lactylation levels in the sclera promote myopia.^481^ This indicates the possible function of the AAV delivery system in targeted gene editing and its subsequent application in epigenetic therapy. Furthermore, LOx-catalyzed lactate depletion activates CRISPR/Cas9-mediated SIRPα genome-editing plasmids, which, when combined with a MOF, are used to promote the conversion of M2 macrophages to M1 macrophages in the breast cancer TME, thereby hinting the therapeutic effect of CRISPR/Cas9-mediated genome-editing.^425,489–491^ In a word, extensive breakthrough upon lactate targeted therapy requires further research into lactate/lactylation biology, related molecular pathways, and associated interactions between consonants of TME to provide foundational support.

### Lactate assisted therapy

While lactate has long been considered a productive substrate for energy metabolism of tumors, recent studies have uncovered conflicting perspectives. Extracellular acidosis impedes one-carbon metabolism crucial for short-lived effector T cells, promoting their differentiation, resilience, and anti-tumor cytotoxicity.^318,473^ Concerning immune checkpoint blockade (ICB) therapy, it was demonstrated that inhibiting LDHA in melanoma mouse model significantly boosted the efficacy of anti-PD-1 therapy. This combined approach effectively disrupted the PD-1/PD-L1 pathway, leading to enhanced pro-inflammatory anti-tumor responses, including heightened infiltration and activity of NK cells and CD8+ cytotoxic T cells, and a decreased frequency of Treg cells.^302^ Additionally, extended study validated the immunotherapy sensitization effect of lactate in vivo. Subcutaneous administration of sodium lactate solution promotes antitumor immunity through CM8 + T cells and sensitizes immunotherapy in CRC, NSCLC and melanoma mouse models.^316^ This suggests that the reprogramming of lactate metabolism in T cells may have a nuanced impact on antitumor immunity, posing a potential dual effect that warrants further exploration. Besides, AlkB homolog 5 (ALKBH5) inhibitor ALK-04 enhances the sensitivity of anti–PD-1 immunotherapy and immune cell recruitment by downregulating MCT4 levels and lactate production in melanoma.^492^ Moreover, anti-APOC2^K70-lac^ antibody interferes with K70 lactylaton of APOC2, thus thwarting FFA release, blocking the accumulation of Treg cells and sensitizing anti-tumor response of immunotherapy.^181^

Regarding lactate-targeted strategies for radiotherapy sensitization, Yao et al. developed a novel CoMnFe-layered double oxides (LDO) nanosheet with multienzyme activities, which not only enhanced ROS production during radiotherapy but also catalyzed lactate into pyruvate. This innovation offers a therapeutic strategy to eliminate lactate from the TME and improving radiotherapy efficacy for uveal melanoma (UM).^493^

In terms of chemotherapy sensitization, lactate has been found to confer chemoresistance by increasing the expression of multidrug resistance-associated protein 1 (MRP1, encoded by ATP-binding cassette sub-family C member 1 (ABCC1)), which induces drug efflux from cells. Nevertheless, the use of NaHCO3 to neutralize lactate may reverse this resistance, making NSCLC cells more susceptible to the chemotherapeutic drug etoposide.^494^ To add up, leveraging the acidity and high levels of lactate in the TME, the engineered LOx-immobilized Ce-benzenetricarboxylic acid (Ce-BTC) MOF facilitates the intratumoral production of hydroxyl radicals (·OH) through a cascade process. This strategy allows for pH-dependent, sensitized and targeted chemotherapy in HCC mouse model.^495^

In addition to immunotherapy, radiotherapy and chemotherapy, lactate-targeted therapy can also have a synergistic effect when combined with targeted therapies. It is demonstrated that lactate stabilized NF-κB within CAFs, prompting the secretion of tumor-promoting HGF, which induced TKIs resistance in tumor cells. When targeted therapies are used in combination with MCT1/2 inhibitor AZD3965 or LDHA inhibitor NHI-Glc-2, the sensitivity is restired in SLCLC and gastric cancer and they achieve more favorable efficacy.^334^ Lactate also induces resistance to pan-Akt inhibitor uprosertib in CRC. Notably, combining uprosertib with MCT1/2 inhibitor AZD3965 reversed this resistance through the inhibition of lactate uptake.^496^

## Conclusion and future directions

Lactate is proven to be a key source of circulating carbohydrates that fuels the TCA cycle and promotes energy production in tumors. Changes in lactate metabolism impact tumor progression, as lactate can enter cells through various pathways, such as intercellular transport via MCT. This process, known as metabolic symbiosis, plays a crucial role in tumor biology by facilitating interactions between different cell populations in the TME. In tumor cells, lactate enhances the lactate shuttle as well as affecting cell signaling pathways, promoting resistance to oxidative stress, and leading to lactylation. In other cell populations, interaction between lactate and immune cells influences cell differentiation, immune response, immune surveillance, and sensitivity to treatment. Additionally, communication between lactate and stromal/endothelial cells reinforces invasiveness and progression of tumors.

Given lactate’s extensive role in cancer metabolism and its influence on both tumor and immune cells, targeting lactate metabolism has emerged as a promising strategy for cancer treatment. When it comes to detection of lactate/lactylation, advanced molecular and imaging techniques are shedding new light on the mechanisms and functional significance of fluctuations in tissue lactate and lactylation levels throughout tumor progression. In addition, researchers have explored various approaches to inhibit lactate production or block its transport within tumors, with the goal of disrupting the metabolic symbiosis that sustains tumor growth. Despite the potential of targeting lactate, most lactate-targeted therapies remain in the preclinical stage, with only a few advancing to clinical trials. One notable example is AZD3965, an inhibitor of MCT1/2, which has undergone a phase I clinical trial (NCT01791595) in patients with advanced cancer. This trial has provided insights into the pharmacokinetics and adverse effects of AZD3965, but further clinical trials are needed to assess its efficacy and therapeutic potential in a broader range of cancers.

However, significant challenges remain in the development of effective lactate/lactylation-targeted therapies. One major obstacle is that determining the quantitative contribution of modulating lactate balance to therapeutic efficacy remains challenging. Besides, according to the metabolic heterogeneity across different tumor types, clinical stages, and distinct cell populations within the TME, the heterogeneity and complexity of the TME make it difficult to achieve effective drug concentrations at the tumor site and complicate the effectiveness of metabolic-targeted drugs. Additionally, when lactate inhibitors are applied to tumors characterized by phenotypic plasticity, they often exhibit high off-target effects and low specificity to lactate metabolism, making it difficult to pinpoint their true pharmacological mechanisms and therapeutic benefits. Moreover, changes in non-cancerous cells during treatment introduce further complexities, as these indiscriminate alterations may undermine the effectiveness of immune response, confront normal physiological functions and lead to unintended side effects.

To tackle the challenges associated with lactate/lactylation-targeted cancer therapies, recent investigations have increasingly focused on several key areas of innovation that hold significant promise for improving treatment outcomes. One major area of focus is the design and optimization of novel small-molecule inhibitors that exhibit enhanced specificity and efficacy against tumor cells. These inhibitors aim to selectively disrupt lactate-related pathways, thereby reducing the cancer cells’ metabolic adaptability. In tandem with this, researchers are developing advanced drug delivery systems that are both robust and efficient, enhancing bioavailability and precision targeting of therapeutic agents. Such innovations are crucial for ensuring that drugs reach their intended sites of action in sufficient concentrations to exert their effects while minimizing side effects on healthy tissues. Furthermore, the exploration of rational, synergistic drug combination strategies is gaining traction. These combination therapies are designed to target multiple pathways simultaneously, thereby minimizing the potential for drug resistance and improving overall therapeutic efficacy. By employing a multi-faceted approach, these strategies aim to overcome the limitations of current therapies, ultimately leading to more favorable treatment outcomes and improved prognoses for cancer patients.^497^

In conclusion, although lactate-targeted therapies show considerable promise in the realm of cancer treatment, they are still in the early stages of development, and substantial efforts are required to unlock their full potential. A comprehensive understanding of how lactate interacts with various metabolic and epigenetic processes in cancer is crucial for creating more effective therapeutic strategies. By elucidating these complex relationships, researchers can develop novel cancer therapies that leverage the unique properties of lactate and lactylation. Such advancements in lactate/lactylation-targeted therapy are urgently needed to effectively suppress tumor growth, overcome drug resistance, and ultimately improve patient outcomes, paving the way for more personalized and successful treatment approaches in oncology.

## Data Availability

Not applicable.
